# Reframing Triazoloacridinone C‑1305 as a G‑Quadruplex Pharmacophore

**DOI:** 10.1021/acs.jpcb.6c01505

**Published:** 2026-05-11

**Authors:** Julia Pakuła, Julia Borzyszkowska-Bukowska, Monika Pawłowska, Rafał Tomaszczyk, Karolina Zielińska, Ewa Paluszkiewicz, Tomasz Laskowski

**Affiliations:** † Department of Pharmaceutical Technology and Biochemistry, Faculty of Chemistry, 49557 Gdańsk University of Technology, Gabriela Narutowicza Str. 11/12, Gdańsk 80-233, Poland; ‡ Institute of Bioorganic Chemistry, Polish Academy of Sciences, Zygmunta Noskowskiego Str. 12/14, Poznań 61-704, Poland

## Abstract

What was once assigned as GGG intercalation can, in retrospect, be read as G-quadruplex recognition. Here, we reframe the clinically investigated triazoloacridinone C-1305 as a high-affinity ligand of the c-MYC promoter G4 model Pu22. UV–Vis speciation resolved four spectral forms and yielded three macroscopic p*K*_a_ values, demonstrating that the dominant protonation state is retained under our NMR conditions and near physiological pH. Two orthogonal titration readouts—imino ^1^H NMR and UV–Vis global analysis—converge on a well-defined 2:1 Pu22:C-1305 complex with submicromolar affinity and a distinctive ligand-induced “destabilize–refold” NMR signature. Variable-temperature NMR reveals pronounced thermal stabilization of Pu22 upon binding. Intermolecular NOEs together with 3D well-tempered metadynamics map a recognition mode dominated by end-stacking on both terminal G-tetrads, with flanking segments forming secondary pockets and enabling alternative bound substates. In cell assays, C-1305 shows low-micromolar cytotoxicity across multiple cancer cell lines while being markedly less toxic to normal cells, outperforming cisplatin in potency. These results reposition C-1305 from a putative GGG intercalator to a G4-targeting pharmacophore and outline a route to side-chain engineering for improved G4 selectivity.

## Graphical abstract

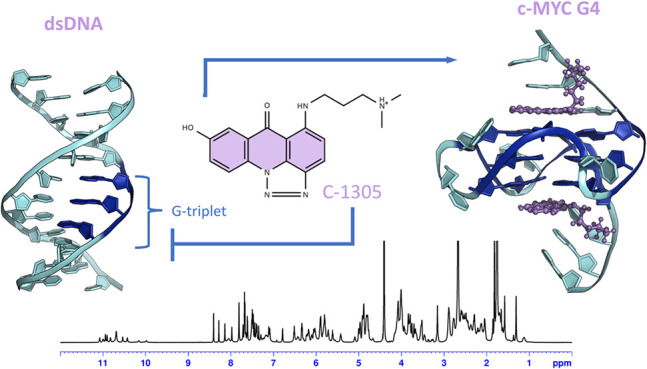

## Introduction

G-quadruplexes (G4s) are noncanonical DNA structures formed within guanine-rich genomic regions. They occur in key genomic loci such as telomeres and promoter elements of selected oncogenes, including c-MYC and k-RAS, where they play important roles in transcriptional regulation.^1,2^ c-MYC overexpression is associated with impaired cellular differentiation, enabling sustained proliferation-one of the hallmarks promoting tumor growth,^3^ and the c-MYC-encoded transcription factor activates genes required for proliferation.^2,4^ In many cancers, the equilibrium between G4 and canonical B-DNA is shifted toward B-DNA, facilitating uncontrolled recruitment of transcription factors.^2,3,5^ Therefore, promoter G4s, acting as a “molecular switch” in gene silencing, constitute attractive molecular targets for anticancer strategies aimed at stabilizing the G4 fold and suppressing transcription.^2,3^ However, recent literature indicates that *MYC* G4 structures can also recruit transcriptional factors and chromatin-binding proteins. Studies emphasize the greater importance of G4 structures themselves than specific DNA sequences. This approach offers the opportunity to broaden the view of G4-stabilizing ligands, not only as silencing element blockers but also as inhibitors competing with transcriptional factors for binding to G4 in promoter regions.^6^

Pu22 is a modified sequence derived from the NHE III region of the P1 promoter of the c-MYC oncogene and is widely used as a model gene-silencer element.^2^ Because the G-quadruplex formed by the native Pu27 sequence is conformationally heterogeneous, a Pu22 variant containing two key G → T mutations (G14/T and G23/T) was introduced to enable high-resolution NMR studies, yielding a single dominant parallel topology in the presence of potassium ions.^3,7^ This sequence is thermally robust and relatively stable under laboratory conditions, making it a convenient model for probing interactions with small molecules, including acridine derivatives.^7^ Stabilization of Pu22 by small ligands is expected to hinder transcription factor binding and thereby reduce oncogene overexpression in cells.^2,3^

Here, we investigate the interaction of Pu22 (Figure 1a) with the acridine derivative 5-[[3-(dimethylamino)­propyl]­amino]-8-hydroxy-6*H*-v-triazolo­[4,5,1-de]­acridin-6-one (C-1305) (Figure 1b). C-1305 is known for its anticancer activity in cell-based studies and has previously been proposed as a guanine-triplet intercalator, largely based on surface plasmon resonance (SPR), DEPC chemical probing, and computational considerations.^8^ In contrast, our NMR studies on dsDNA revealed no observable preference for intercalation into GGG tracts; instead, C-1305 consistently favors purine–pyrimidine steps, with the TA/TA step bound most strongly and GG/CC most weakly, in apparent disagreement with earlier expectations.^9^ These findings motivated us to test whether C-1305 may instead recognize and stabilize promoter G-quadruplex DNA, thereby reframing triazoloacridinone C-1305 as a G4-targeting pharmacophore. Notably, C-1305 was reported to display increased cytotoxicity in PARP-1-deficient contexts—consistent with a mechanism in which a higher cellular G4 load enhances the impact of ligand-mediated G4 stabilization.^8^

**Figure 1:**
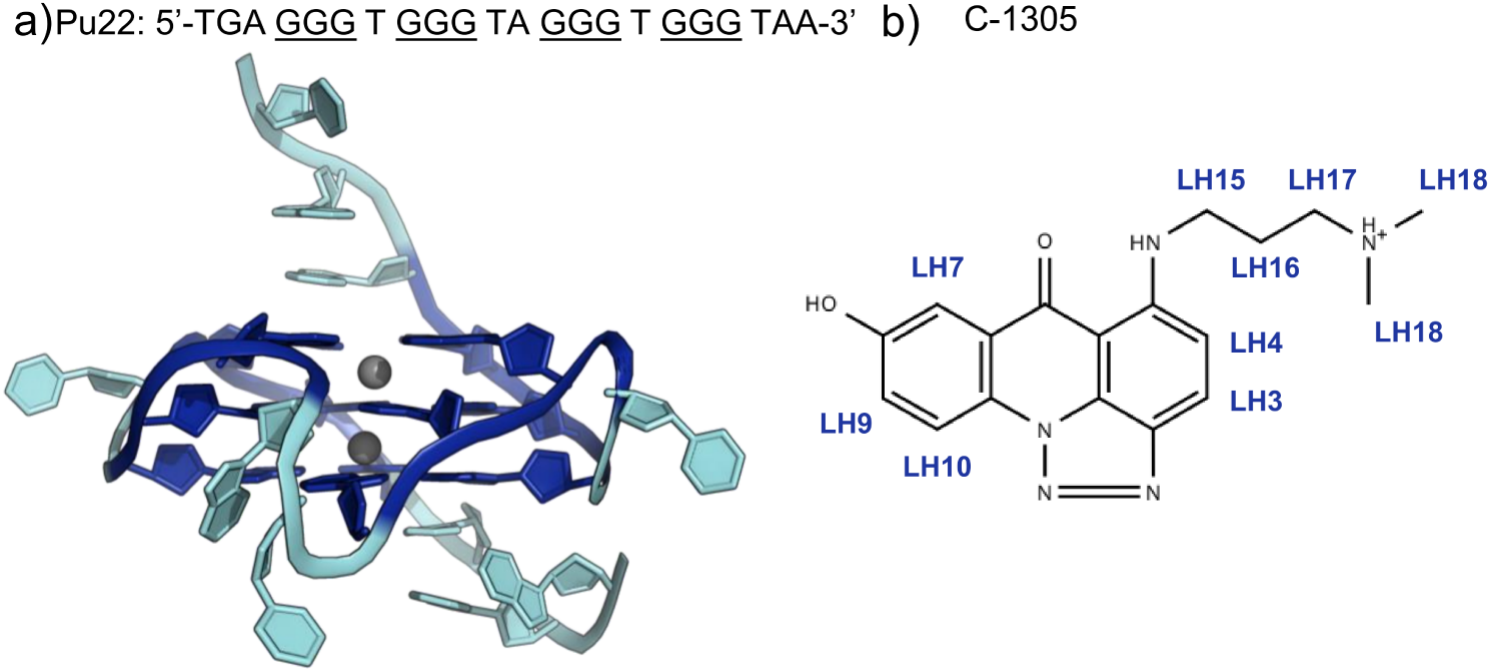
G-quadruplex and ligand structures. a) The sequence and structure of Pu22 G-quadruplex. Guanines forming G-tetrads are shown in blue, while adenines, thymines, and guanines not arranged in G-tetrads are shown in cyan. Potassium ions are shown in gray. b) The chemical structure of 5-[[3-(dimethylamino)­propyl]­amino]-8-hydroxy-6*H*-v-triazolo­[4,5,1-de]­acridin-6-one, code name C-1305, with the atom numbering used in this study.

## Materials and Methods

### DNA and Ligand Sample Preparation for NMR

C-1305 was synthesized at Gdańsk University of Technology. A stock solution of C-1305 was prepared in deionized water (10 mM) and further diluted as required for UV–Vis titrations and DNA complexation experiments.

The Pu22 oligonucleotide with the sequence 5′-TGA GGG T GGG TA GGG T GGG TAA-3′ (Metabion GmbH, Germany) was washed with 4 M LiCl and subsequently rinsed several times with deionized water to remove residual triethylammonium acetate (TEAA, remaining from the manufacturer’s purification procedure) and other unwanted ions. Prior to complex formation, Pu22 was thermally annealed by heating to ∼90 °C and then cooling to room temperature.

For NMR experiments, Pu22 samples contained either 4 OD (∼17 nmol) or 20 OD (∼85 nmol) of DNA, dissolved in 10 mM potassium cacodylate buffer (pH 5.0) supplemented with 10 mM KCl. The total sample volume was 200 μL, and the solvent composition was H_2_O/D_2_O (9:1, v/v). Solutions containing 4 OD of Pu22 were used for 1D ^1^H NMR monitoring of DNA/ligand complex formation, whereas solutions containing 20 OD were employed for 2D NMR measurements.

### NMR Spectroscopy

NMR spectra were recorded on a Bruker Avance III HD 700 MHz spectrometer equipped with a QCI CryoProbe installed at the Institute of Bioorganic Chemistry, Polish Academy of Sciences, Poznań, Poland. Spectra were processed using Bruker TopSpin 4.5.0 software and NMRFAM-SPARKY.^10^ Measurements were performed in a H_2_O/D_2_O (9:1, v/v) and 100% D_2_O solvent system. Chemical shifts (δ) are reported in parts per million (ppm), referenced to the residual proton signal of H_2_O at δ_H_ = 4.610 ppm.

One-dimensional ^1^H NMR spectra were performed at 45 °C. ^1^H NMR spectra of melting experiment were acquired over a temperature range from 5 to 75 °C. Reference NMR spectra for Pu22 consisted of NOESY (150 and 400 ms mixing time) and TOCSY (60 ms spin-lock time), which were conducted at 45 °C in H_2_O/D_2_O (9:1, v/v) solvent system. Additionally, NOESY (400 ms mixing time), TOCSY (60 ms spin-lock time), and ed-HSQC were performed at 45 °C in pure D_2_O (Table S1).

Pu22/C-1305 complex two-dimensional ^1^H–^1^H spectra were recorded with a spectral width of 6313 Hz. NOESY spectra were recorded with a mixing time of 400 ms in a 2048 × 512 matrix, using 128 scans per increment with a spectral width of 9803 Hz (Table S2, Figures S1–S5). Experiments were acquired at 45 °C in H_2_O/D_2_O (9:1, v/v) solvent system.

### Cell Cultures

In the study, six human cancer cell lines were used: pancreatic cancer cell lines Panc-1 and MIA PaCa-2, nonsmall cell lung cancer (NSCLC) cell lines H460 and A549, colorectal cancer cell line HCT116, and triple-negative breast cancer cell line MDA-MB-231. All cell lines were purchased from the American Type Culture Collection (ATCC, Manassas, VA, USA). Panc-1, MIA PaCa-2, and MDA-MB-231 cells (H460, A549, Panc-1, HCT116, MDA-MB-231) were maintained in high-glucose Dulbecco’s modified Eagle’s medium (DMEM HG, Merck, Darmstadt, Germany), H460 cells in RPMI 1640 medium (Merck, Darmstadt, Germany), A549 cells in Ham’s F-12K medium (Kaighn’s modification of F-12) (Merck, Darmstadt, Germany), while HCT116 cells were maintained in McCoy’s 5A medium. All media contained 2 mM l-glutamine and were supplemented with 10% fetal bovine serum (FBS, Merck, Darmstadt, Germany), 100 U/mL penicillin, and 100 μg/mL streptomycin (mixture, Merck, Darmstadt, Germany). Cells were incubated in a humidified atmosphere of 5% CO_2_ at 37 °C. All experiments were performed on cells in a logarithmic growth phase.

### Cell Growth Inhibition Assay

To investigate the effect of C-1305 on cell viability, a colorimetric analysis was performed using MTT (3-(4,5-dimethyl-2-thiazolyl)-2,5-diphenyl-2*H*-tetrazolium bromide, Merck) based on the reduction of this yellow salt in metabolically active cells to purple formazan crystals. Panc-1, MIA PaCa-2 (5,000/well), H460, A549 (2,500/well), and HCT116 cells (1,000/well) were seeded in 96-well plates, and the following day, the tested C-1305 was added at concentrations up to 50 μM. Stock solutions (10 mM) were prepared in sterile water, as well as dilutions. 2 μL of each solution was added to 198 μL of cell medium. Cells were incubated with drugs for 72 h. MTT solution (4 mg/mL) was added for 3 h, formazan crystals were dissolved in DMSO, and absorbance was measured at 540 nm using a microplate reader (Bio-Rad, Hercules, CA, USA). The concentration of the drug required to inhibit cell growth by 50% (IC_50_) compared to untreated control cells was determined from the curves plotting survival as a function of dose. Each assay was performed at least 4 times.

### UV–Vis Spectroscopy

#### General Information

All UV–Vis spectra were recorded on a SPECORD 200 Plus spectrophotometer (Analytik Jena) equipped with a thermostated cell holder. Measurements were performed in 1 cm path-length quartz cuvettes using a double-beam configuration. Spectra were collected with a wavelength step of 0.1 nm at a scan speed of 10 nm s^–1^. All measurements were temperature-controlled; after the sample reached the target temperature, an additional 30 s equilibration period was applied prior to data acquisition. For each experiment, an appropriate reference solution was placed in the reference compartment throughout the measurement.

### UV–Vis p*K*_a_ Series

UV–Vis spectra of C-1305 were recorded to determine its acid–base dissociation constants (p*K*_a_) over the pH range of 1.0–10.0 in 0.5 pH-unit increments. The pH was adjusted using hydrochloric acid solutions (pH 1.0–3.0), acetate buffer (pH 3.0–5.5), phosphate buffer (pH 6.0–7.5), and borate buffer (pH 8.0–10.0). All solutions were prepared at a constant ionic strength of 10 mM. The concentration of C-1305 was 10 μM in every sample. UV–Vis spectra were acquired in the 350–550 nm wavelength range with a 0.1 nm step size at 37 °C (temperature-controlled).

### UV–Vis Titration

The initial ligand solution had an established concentration of 10 μM and a volume of 1.00 mL and was prepared in 10 mM potassium cacodylate buffer (pH 5.0) supplemented with 10 mM KCl. The DNA stock solution (prepared in water) was adjusted such that 10 μL of the DNA stock corresponded to 1 mol equiv of DNA relative to the ligand amount present in the cuvette. Aliquots of the DNA stock were added stepwise to the ligand solution to reach sequential DNA equivalents *n* = 0.1, 0.15, 0.2, 0.3, 0.5, 0.7, 1.0, 1.5, 2, 3, 5, and 7 (relative to ligand). In total, 70 μL of the DNA stock was added, and the resulting volume increase (from 1.00 to 1.07 mL) was explicitly accounted for in the calculations. After each addition, a UV–Vis spectrum was recorded in the 350–550 nm range with a 0.1 nm step size at 45 °C (conditions matching those used in the NMR experiments). A spectrum of the free ligand (*n* = 0) was also recorded.

### Chemometric Analysis

#### General Information on Principal Component Analysis (PCA)

The section documenting the in-house R^11^ workflow used to perform principal component analysis (PCA) of UV–Vis spectra, diagnose data structure, and generate residual (unexplained) spectra for successive reconstruction ranks can be found in Supporting Information.

### UV–Vis p*K*_a_ Determination (C-1305)

#### Acid–Base Model and Theoretical Molar Fractions

The pH-dependent spectra were decomposed into *M* spectrally distinct components (*M* = 4), which were interpreted as acid–base forms of the ligand in equilibrium. The model assumes sequential deprotonation steps characterized by stepwise dissociation constants *K*_a,i_ (equivalently p*K*_a,i_ = −log_10_ *K*_a,i_). For a system with *m* dissociation steps (*m* = *M* – 1), the species are denoted L H_*m*_, L H_{*m*–1}_,..., L (where L H_*m*_ is the most protonated form).

Let *h* = [H^+^] = 10^–pH^ and *K*_a,i_ = 10^–p*K*_a_,*i*^. The binding (distribution) polynomial for the *m*-step acid–base equilibrium is eqs 1 and 2:

1 $$Q\left(h\right)=h^{m}+K_{a,1}h^{m-1}+K_{a,1}K_{a,2}h^{m-2}+\cdot \cdot \cdot +\overset{m}{\underset{i=1}{\prod }}K_{a,i}$$

2 $$\alpha_{0}=\frac{h^{m}}{Q\left(h\right)},,\alpha_{1}=\frac{K_{a,1}h^{m-1}}{Q\left(h\right)},,\alpha_{2}=\frac{K_{a,1}K_{a,2}h^{m-2}}{Q\left(h\right)},...,\alpha_{m}=\frac{\overset{m}{\underset{i=1}{\prod }}K_{a,i}}{Q\left(h\right)}$$

The coefficients α_*j*_ represent the theoretical molar fractions of the *M* forms at a given pH and satisfy their sum = 1 at every pH. In the implementation, the numerators are evaluated in log_10_ space via pH and p*K*_a_ values using *h* = 10^–pH^ and *K*_a,i_ = 10^–p*K*_a_,i^.

For *M* = 4 (three p*K*_a_ values, *m* = 3), the fractions are (eqs 3, 4)­

3 $$Q\left(h\right)=h^{3}+K_{a,1}h^{2}+K_{a,1}K_{a,2}h+K_{a,1}K_{a,2}K_{a,3}$$

4 $$\alpha_{0}=\frac{h^{3}}{Q\left(h\right)},,\alpha_{1}=\frac{K_{a,1}h^{2}}{Q\left(h\right)},,\alpha_{2}=\frac{K_{a,1}K_{a,2}h}{Q\left(h\right)},,\alpha_{3}=\frac{K_{a,1}K_{a,2}K_{a,3}}{Q\left(h\right)}$$

### Objective Function and Parameter Estimation

Experimental molar fractions (obtained from spectral decomposition) are denoted α^exp^_*j*_(pH_*i*_). Model parameters (p*K*_a_ values) were estimated by nonlinear least-squares by minimizing the sum of squared errors (SSE) over all pH points and all components (eq 5):

5 $$\mathrm{SSE}=\overset{N}{\underset{i=1}{\sum }}\overset{m}{\underset{j=0}{\sum }}{\left(\alpha_{j}^{\exp}\left(pH_{i}\right)-\alpha_{j}^{\mathrm{th}}\left(pH_{i}\right)\right)}^{2}$$

Optimization was performed using the L-BFGS-B algorithm with box constraints on p*K*_a_ values (0–10 in the scripts), starting from user-provided initial guesses. To reduce ambiguity due to arbitrary component ordering in spectral decomposition, the columns of the experimental fraction matrix were reordered prior to fitting by sorting components according to the pH position of their maxima (from low to high pH).

### Cross-Validation and Visualization

Model robustness was evaluated using leave-one-out (LOO) cross-validation: each pH point was excluded in turn, parameters were refitted to the remaining points, and the mean ± standard deviation of p*K*_a_ values was reported. Final plots show experimental points and theoretical curves as a function of pH; vertical dashed lines indicate the optimized p*K*_a_ values and serve as visual guides.

### UV–Vis Titration Analysis—2-Site DNA-Ligand Binding Model

#### Model Definition and Assumptions

We consider a DNA receptor, D, that presents two ligand-binding sites, A and B (5′-side and 3′-side of the G-quadruplex). The ligand L binds to each site with microscopic association constants *K*_a,A_ and *K*_a,B_ (equivalently, dissociation constants *K*_d,A_ = 1/*K*_a,A_ and *K*_d,B_ = 1/*K*_a,B_). Double occupancy (DL2) is allowed. Spectroscopically, the singly bound states, DLA and DLB, are treated as indistinguishable and therefore pooled into a single UV–Vis component denoted DL.

Microscopic equilibria (association constants—eqs 6–9)­

6 $$D+L⇌\mathrm{DLA},K_{a,A}=\frac{\left[\mathrm{DLA}\right]}{\left[D\right]\left[L\right]}$$

7 $$D+L⇌\mathrm{DLB},K_{a,B}=\frac{\left[\mathrm{DLB}\right]}{\left[D\right]\left[L\right]}$$

8 $$\mathrm{DLA}+L⇌\mathrm{DL}2,K_{a,B}^{\left(2\right)}=\omega K_{a,B}$$

9 $$\mathrm{DLB}+L⇌\mathrm{DL}2,K_{a,A}^{\left(2\right)}=\omega K_{a,A}$$

Here, ω is a (dimensionless) cooperativity factor. ω = 1 corresponds to independent sites. ω > 1 indicates positive cooperativity (the second binding event is enhanced), while ω < 1 indicates negative cooperativity.

Binding polynomial (partition function) as a function of free ligand concentration *L* = [L] (eq 10):

10 $$Q\left(L\right)=1+K_{a,A}L+K_{a,B}L+\omega K_{a,A}K_{a,B}L^{2}$$

Fractions of DNA receptor in each microstate are obtained directly from *Q*(*L*) (eqs 11–14):

11 $$f_{0}=\frac{\left[D\right]}{D_{t}}=\frac{1}{Q\left(L\right)}$$

12 $$f_{A}=\frac{\left[\mathrm{DLA}\right]}{D_{t}}=\frac{K_{a,A}L}{Q\left(L\right)}$$

13 $$f_{B}=\frac{\left[\mathrm{DLB}\right]}{D_{t}}=\frac{K_{a,B}L}{Q\left(L\right)}$$

14 $$f_{2}=\frac{\left[DL2\right]}{D_{t}}=\frac{\omega K_{a,A}K_{a,B}L^{2}}{Q\left(L\right)}$$

Therefore, for a given total DNA concentration *D_t_*, the species concentrations are (eq 15):

15 $$\left[D\right]=D_{t}f_{0},,\left[\mathrm{DLA}\right]=D_{t}f_{A},,\left[\mathrm{DLB}\right]=D_{t}f_{B},,\left[\mathrm{DL}2\right]=D_{t}f_{2}$$

### Ligand Mass Balance and Numerical Solution for Free Ligand

The UV–Vis decomposition yields ligand-centered molar fractions (the three spectral forms sum to 1 with respect to the ligand population). Thus, we solve the ligand mass balance (eq 16):

16 $$L_{t}=\left[L\right]+\left[\mathrm{DLA}\right]+\left[\mathrm{DLB}\right]+2\left[\mathrm{DL}2\right]$$

Substituting the expressions above and using *Q*(*L*) gives a single scalar equation for *L* = [L] (eq 17):

17 $$L_{t}=L+D_{t}\frac{\left(K_{a,A}+K_{a,B}\right)L+2\omega K_{a,A}K_{a,B}L^{2}}{Q\left(L\right)}$$

For each titration point, this equation is solved numerically for *L* in the interval [0, *L_t_*] (using a robust 1D root-finder).

### Theoretical Ligand Fractions Compared to Experimental UV–Vis Fractions

Once *L* is obtained, the theoretical ligand-centered molar fractions used for fitting are (eqs 18–20)­

18 $$\chi_{0}=\chi_{\mathrm{freeL}}=\frac{L}{L_{t}}$$

19 $$\chi_{1}=\chi_{\mathrm{DL}}=\frac{\left[\mathrm{DLA}\right]+\left[\mathrm{DLB}\right]}{L_{t}}=\frac{D_{t}\left(K_{a,A}+K_{a,B}\right)L}{Q\left(L\right)L_{t}}$$

20 $$\chi_{2}=\chi_{\mathrm{DL2}}=\frac{2\left[\mathrm{DL}2\right]}{L_{t}}=\frac{2D_{t}\omega K_{a,A}K_{a,B}L^{2}}{Q\left(L\right)L_{t}}$$

By construction, χ_0_ + χ_1_ + χ_2_ = 1 for every titration point (up to numerical precision). These χ values are fitted to the experimentally determined fractions (ratios) by minimizing the sum of squared errors across all titration points and all three forms.

### Parameter Estimation

Parameters are optimized by nonlinear least-squares: we minimize the objective function (eq 21)­

21 $$\mathrm{SSE}=\overset{N}{\underset{i=1}{\sum }}\overset{2}{\underset{k=0}{\sum }}{\left(\chi_{k,i}^{\exp}-\chi_{k,i}^{\mathrm{th}}\right)}^{2}$$

with respect to *K*_d,A_ and *K*_d,B_ (and optionally ω) using the Nelder–Mead algorithm. To enforce positivity, optimization is carried out in log-parameters (e.g., log *K*_d,A_, log *K*_d,B_, log ω). A multistart strategy (random jitter around an initial guess) is used to reduce the risk of local minima.

### Model Selection

In the main manuscript, we report the minimal model with ω fixed to 1 (independent sites), while the ω-fit model is discussed in the Supporting Information.

### Derived Macroscopic Constants

For convenience and for comparison with macroscopic (stepwise) binding constants, we also report the binding-polynomial coefficients (eqs 22, 23):

22 $$\beta_{1}=K_{a,A}+K_{a,B}$$

23 $$\beta_{2}=\omega K_{a,A}K_{a,B}$$

The corresponding macroscopic stepwise association constants are (eqs 24–27)­

24 $$K_{1}^{\mathrm{macro}}=\beta_{1}$$

25 $$K_{2}^{\mathrm{macro}}=\frac{\beta_{2}}{\beta_{1}}$$

26 $$K_{d,1}^{\mathrm{macro}}=\frac{1}{K_{1}^{\mathrm{macro}}}$$

27 $$K_{d,2}^{\mathrm{macro}}=\frac{1}{K_{2}^{\mathrm{macro}}}=\frac{\beta_{1}}{\beta_{2}}$$

In addition, the conditional microscopic dissociation constants for the second binding event are (eqs 28, 29)­

28 $$K_{d,2}\left(A\to B\right)=\frac{K_{d,B}}{\omega }$$

29 $$K_{d,2}\left(B\to A\right)=\frac{K_{d,A}}{\omega }$$

### Software

All analyses were performed using custom scripts written in the R programming language^12^ and executed in RStudio.^13^ Parameter estimation relied on base R numerical routines (stats:optim­() with method = “L-BFGS-B” or “Nelder–Mead”, uniroot­() and standard plotting functions). Additional R packages (mdatools,^14^ pracma,^15^ matlib,^16,17^ signal^18^) were used only for general utilities where appropriate. The full scripts are available from the authors upon reasonable request.

### Molecular Modeling Calculations

#### General Information on Molecular Modeling

All molecular simulations were carried out in the NPT ensemble using the GAFF2 ^19^ force field and GROMACS 2023.2 ^20,21^ coupled to the PLUMED 2.10 plugin.^22^ Periodic boundary conditions were applied in all three dimensions. The temperature was maintained at 318 K using the velocity-rescale thermostat,^23^ while the pressure was kept at 1 bar using the C-rescale barostat.^24^ Long-range electrostatic interactions were treated using the particle-mesh Ewald (PME) method^25^ with a real-space cutoff of 12 Å. Van der Waals interactions were truncated at 12 Å and smoothly switched from 10 Å. The equations of motion were integrated using the Verlet algorithm^26^ with a time step of 2 fs.

### 2D NMR Data Refinement

The structure of ligand C-1305 was built de novo in Avogadro v1.95.1 ^27^ and subjected to an initial geometry optimization (“straightening” of the molecular scaffold) using OpenBabel v3.1.0 with the MMFF94s force field.^28^ Based on the experimentally determined p*K*_a_ values and to match the conditions of the NMR experiments (pH 5.0), C-1305 was modeled with a nonionized phenolic group and a protonated tertiary amine in the side chain.

The ligand was subsequently parameterized using GAFF2 (Generalized Amber Force Field, v2) as implemented in AmberTools 23.6 (ambermd.org). The parameters were further refined by recalculating partial atomic charges from ab initio computations at the MN12SX/6-31G* level using Gaussian 16 (g16.c02)^29^ installed on the Tryton supercomputer (TASK, Gdańsk). The Pu22 G-quadruplex structure was retrieved from the Protein Data Bank (PDB ID: 2L7V) and described with the ff99SB + parmbsc1 force field.

One Pu22 molecule and two C-1305 molecules were placed in proximity (one ligand at the 5′ side and one at the 3′ side of the G-quadruplex) using VMD v2.0.0.5a.^30^ The system was solvated in a periodic dodecahedral box of TIP3P water. To reproduce the ionic conditions of the NMR measurements, the system was neutralized with K^+^ ions and supplemented with additional KCl to reach an overall ionic strength of 20 mM. All simulations were performed using GROMACS 2023.2 with the PLUMED 2.10 plugin. The solvated system was equilibrated in the NVT ensemble, followed by NPT equilibration, applying positional restraints on heavy atoms during the equilibration stages.

After equilibration, NOE-derived DNA/ligand distance restraints were imposed using the distance-restraint functionality of GROMACS (an additional penalty potential is applied when an interatomic distance exceeds the specified bounds). Based on the measured NOESY cross-peak volumes, restraints were classified into three categories: strong (0.200–0.325 nm), medium (0.250–0.450 nm), and weak (0.300–0.600 nm). The restraint strength assigned to each atom pair followed the classification reported in Table S3. Notably, reliable conversion of NOE volumes into averaged interproton distances was not feasible due to the long NOESY mixing time (400 ms); under these conditions, the NOE buildup is no longer in the initial linear regime, and the observed intensities cannot be assumed to scale linearly with *r* ^–6^.

The resulting system was subjected to restrained molecular dynamics for 1 μs. A representative structure from the most populated structural cluster (72.6% of the trajectory) is shown in Figure 3A of the main manuscript.

### 3D Well-Tempered Metadynamics Calculations

#### General Philosophy

The Pu22 G-quadruplex comprises two external G-tetrads which—based on prior NMR evidence—were considered the most favorable binding sites for monoacridines in this system. Accordingly, two simulation systems were defined for C-1305 that differed only in which external tetrad was preoccupied by a restrained ligand molecule, i.e., the 3′ or the 5′ face of the G4.

In both systems, the simulation box contained one Pu22 molecule and two molecules of C-1305. One ligand was positioned in the vicinity of a chosen external G-tetrad (approximately 0.7 Å from its center of mass) and kept effectively immobilized at that site, whereas the opposite external tetrad was left unoccupied and thus available for binding by the second ligand molecule, which remained freely diffusing and explored the accessible space throughout the simulation.

The immobilization of the “trapped” ligand was enforced by a distance restraint applied between the ligand and the center of mass of the occupied G-tetrad, maintaining a target distance of AT = 0.370 nm with a force constant of KAPPA = 239 kcal mol^–1^ nm^–2^ for the entire simulation time. In the 3′-preoccupied system, the restrained ligand occupied the 3′ external G-tetrad, and the 5′ external G-tetrad remained available for binding of the freely moving ligand. Conversely, in the 5′-preoccupied system, the restrained ligand occupied the 5′ external G-tetrad, while the 3′ external G-tetrad remained accessible to the freely diffusing ligand.

### System Preparation

The two simulation systems were constructed using the preparameterized molecular models described in the preceding subsection. Each system contained one Pu22 G-quadruplex and two molecules of the same ligand (C-1305). The complexes were placed in a rhombic dodecahedral periodic simulation box and solvated with TIP3P water.

To reproduce the ionic conditions of the NMR experiments (10 mM potassium cacodylate buffer supplemented with 10 mM KCl), each system was supplemented with K^+^ and Cl^–^ ions at 0.02 M for each ion type. Thus, the simulation environment effectively corresponded to an aqueous 0.02 M KCl solution.

For each of the two systems, 10 independent starting frames were generated. These frames differed only in the initial position of the “mobile” ligand in the simulation box, while the rest of the system remained unchanged. The starting positions were chosen to be distributed as uniformly as possible around the central G-quadruplex, ensuring that, in each frame, the mobile ligand approached Pu22 from a different direction. A single set of 10 ligand placements was prepared and then propagated identically to both systems using an in-house Python script. In total, 20 starting frames were generated (10 for the system with the 3′ tetrad occupied and 10 for the system with the 5′ tetrad occupied).

### Reaction Coordinates

To efficiently map the free-energy landscape of the investigated complex, three-dimensional well-tempered metadynamics (3D-WT-MTD) was employed. The simulations were performed using three reaction coordinates (RCs), denoted ζ, ρ, and ϕ:

- ζ (radial distance): The distance between the center of mass (COM) of the middle G-tetrad and the COM of the ligand. The COM of the middle G-tetrad was defined using four nitrogen atoms located at the corners of the tetrad, each taken from a different guanine at the glycosidic linkage position (i.e., the atom connecting the base to the sugar). The ligand COM was defined using two para-related carbon atoms within the central six-membered ring of C-1305.
- ρ (horizontal angle): A “horizontal” (planar) angle describing the position of the ligand relative to the middle G-tetrad plane; specifically, the angle between the plane defined by the four tetrad nitrogen atoms and the vector connecting the tetrad COM to the ligand COM.
- ϕ (clock/azimuthal angle): A “clock” angle describing the ligand’s azimuthal position around the G-quadruplex. This angle was defined by three points: (i) the glycosidic nitrogen atom of the guanine belonging to the middle G-tetrad that corresponds to the fifth nucleotide when counting from the 3′ end, (ii) the COM of the middle G-tetrad, and (iii) the COM of the ligand.

For each of the two simulation systems (with the immobilized ligand occupying either the 3′ or the 5′ external G-tetrad), a multiple-walker 3D-WT-MTD protocol was used with 10 walkers per system (20 walkers in total). The bias factor was set to 10, and the reference temperature was 318 K (matching the NMR experimental conditions). Gaussian bias potentials with a width of 0.2 Å and an initial height of 0.1 kJ·mol^–1^ were deposited for all CVs every 10 ps. For each system, metadynamics simulations were run for 1000 ns (per walker).

### Cluster Analysis

Clustering of the C-1305 3′-side and C-1305 5′-side simulation systems was performed using the Daura algorithm with an RMSD cutoff of 0.35 nm. Guided by the free-energy surfaces obtained for the ζ pair, the regions of the reaction-coordinate space corresponding to local and global free-energy minima were delineated, as stated in Table S4.

## Results and Discussion

### p*K*_a_ Determination via UV–Vis

Because the optical response and binding behavior of C-1305 can depend on its protonation state, we first established its acid–base equilibria by UV–Vis spectroscopy over a pH range of 1.0–10.0 (Figure 2A). Principal component analysis revealed four spectrally distinct forms, and model fitting with cross-validation yielded three macroscopic p*K*_a_ values (p*K*_a1_ = 1.59 ± 0.05, p*K*_a2_ = 7.87 ± 0.04, p*K*_a3_ = 9.49 ± 0.05), assigned to the triazole ring, the tertiary amine in the side chain, and the phenolic group, respectively (Figure 2B, Figures S7–S11). Importantly, these equilibria indicate that under the NMR binding conditions (pH 5.0) and near physiological pH, the same dominant protonation state prevails, supporting the direct relevance of the present binding analysis to in vivo-like conditions.

**Figure 2:**
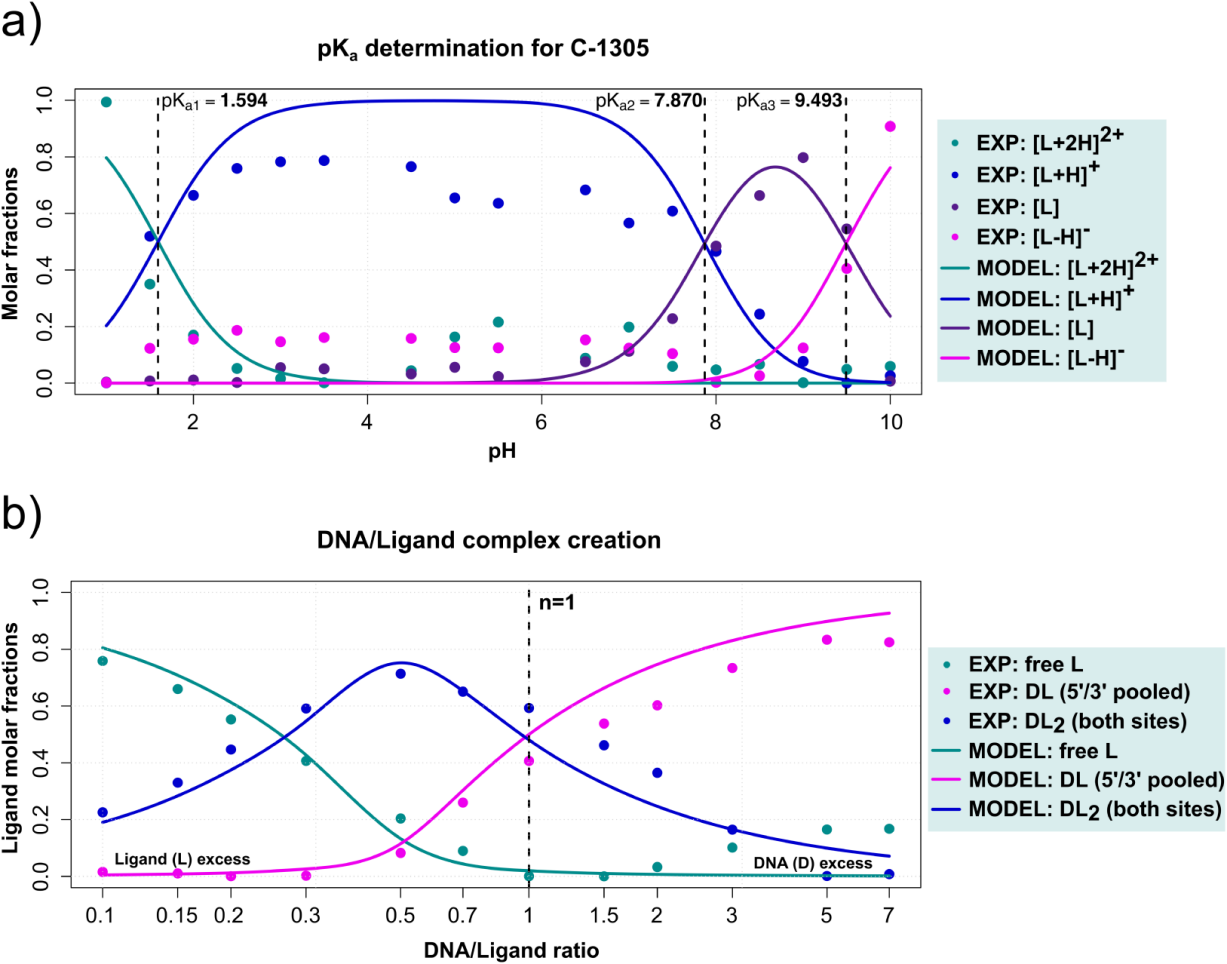
Protonation equilibria of C-1305 and UV–Vis quantification of Pu22 binding. a) UV–Vis pH titration of C-1305 analyzed by PCA and equilibrium-model fitting, revealing four spectral forms and three macroscopic p*K*_a_ values. b) UV–Vis titration of C-1305 with Pu22 (350–550 nm) with global spectral decomposition and least-squares fitting to a minimal two-site binding model (dilution-corrected; see Supporting Information).

### One-Dimensional NMR and UV–Vis Titration

We then established Pu22 binding and stoichiometry using two orthogonal titration readouts (Figures 3B and 2B). Monitoring the imino-proton region by 1D ^1^H NMR during titration of Pu22 with C-1305 revealed an unusual “destabilize–refold” behavior: after the first ligand addition, imino signals became undetectable, whereas subsequent additions gradually restored and sharpened the imino resonances into a spectrum clearly distinct from free Pu22 (Figure 3B). This behavior identified an optimal stoichiometric ratio of DNA:ligand = 1:2, consistent with two dominant binding events (Figure 3A). In parallel, UV–Vis titrations (350–550 nm) were analyzed by spectral decomposition into three components, yielding ligand fractions χ_i(n)_ that sum to unity at each DNA equivalent *n*, and the profiles were fitted by least-squares using a minimal two-site binding model for a DNA target with two nonequivalent binding sites, while treating the two singly bound microstates as spectrally indistinguishable (DL_A_ + DL_B_) (Figure 2B). Dilution upon DNA addition was explicitly accounted for (Supporting Information). The independent-sites model (ω = 1) provided a robust fit and stable parameters under leave-one-out cross-validation, giving *K*_d,A_ ≈ *K*_d,B_ ≈ 0.202 ± 0.038 μM; macroscopic binding constants were established as β_1_ = 0.962 μM and β_2_ = 0.241 μM. An extended cooperative model (ω ≠ 1) produced poorly identifiable microscopic parameters and was therefore not used (Supporting Information).

**Figure 3:**
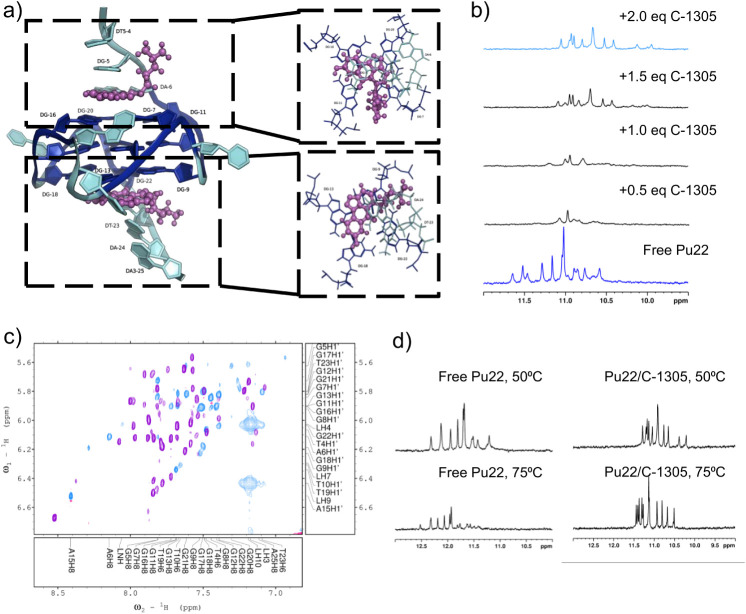
NMR spectroscopy study on the noncovalent adduct. a) The structure of Pu22/C-1305 complex based on NMR spectroscopy distance restraints. b) ^1^H NMR spectra of the titration of Pu22 with C-1305 at pH = 5.0; temperature 45 °C; H_2_O/D_2_O 9:1 v/v in 10 mM cacodylate buffer with 10 mM KCl. The imino proton region is shown. c) The expanded H1′-H8/H6 region of the 2D NOESY spectrum of Pu22 (shown in purple) and Pu22/C-1305 complex (shown in blue). d) Thermal stabilization of Pu22 G-quadruplex. The comparison of thermal stability of free Pu22 (on the left) and Pu22/C-1305 complex (on the right).

Having established 2:1 binding by two independent titration approaches, we next asked whether C-1305 stabilizes the promoter G4 fold. Variable-temperature ^1^H NMR experiments provided direct evidence for ligand-induced thermal stabilization (Figure 3D). Across the 5–75 °C range, the imino signals of free Pu22 broadened markedly at elevated temperatures, consistent with the loss of a persistent folded state under these conditions. In contrast, the Pu22/C-1305 complex maintained sharp imino resonances with no major loss of intensity or substantial chemical-shift drift, demonstrating pronounced stabilization upon ligand binding (Figure 3D).

To elucidate the binding mode underlying this stabilization, we assigned resonances of free Pu22 (Table S1) and the Pu22/C-1305 complex (Table S2) using NOESY (Figure S1–S4), TOCSY, and HSQC (Figure S5) experiments and inspected intermolecular NOEs (Figure 3C). Numerous ligand–G4 contacts were observed (Figure S4), consistent with the triazoloacridinone chromophore adopting an end-stacked orientation relative to terminal G-tetrads. In particular, NOEs between aromatic protons of C-1305 and guanine residues at both the 3′ and 5′ tetrads (e.g., cross-peaks listed as 9 and 1, 2, 6, 7 in Table S3) support π-stacking on terminal tetrads, while additional NOEs to flanking residues (e.g., 1, 5, 8, 10, 11 in Table S3) point to a structured “pocket-like” environment at least at one end of the G4 (Figure 3C). Moreover, NOEs involving the C-1305 side chain and protons in the central tetrad/loop region (12, 19, 21, 23, and 26, 27 in Table S3) suggest that at least one ligand adopts a side-chain orientation approximately perpendicular to the tetrad planes, extending along the G4 surface. The overall density and intensity pattern of contacts indicates a preferred binding site consistent with promoter-G4 ligand paradigms reported for related systems.

### Molecular Dynamics Simulations

Because NMR reports a time-averaged ensemble, we complemented these data with 3D well-tempered metadynamics (3D-WT-MTD) simulations of Pu22 in the presence of two C-1305 molecules (Figure 4, Figure S6). Two independent setups were analyzed, with one ligand prepositioned at either the 3′ or 5′ terminal tetrad, while the second ligand explored binding as a function of three reaction coordinates (two angles and the distance to the G4 center). The resulting free-energy landscapes show no strong preference for groove binding; instead, global minima (Δ*G* ∼ 3.0–3.6 kcal/mol) correspond to end-stacked conformations on terminal tetrads at both ends of Pu22 (Figure 4). Importantly, the 5′ (TGA) and 3′ (TAA) flanking segments contribute to stabilizing low-energy bound states by forming secondary “binding pockets”, rationalizing the prominent flanking contacts observed by NOESY. Clustering further suggested the presence of ligand dimers at either end and alternative configurations in which one ligand remains end-stacked while the second engages a single-stranded flanking segment, including an intercalation-like motif between bases consistent with the experimentally observed preference of C-1305 for 5′-pyrimidine-purine-3′ steps.

**Figure 4:**
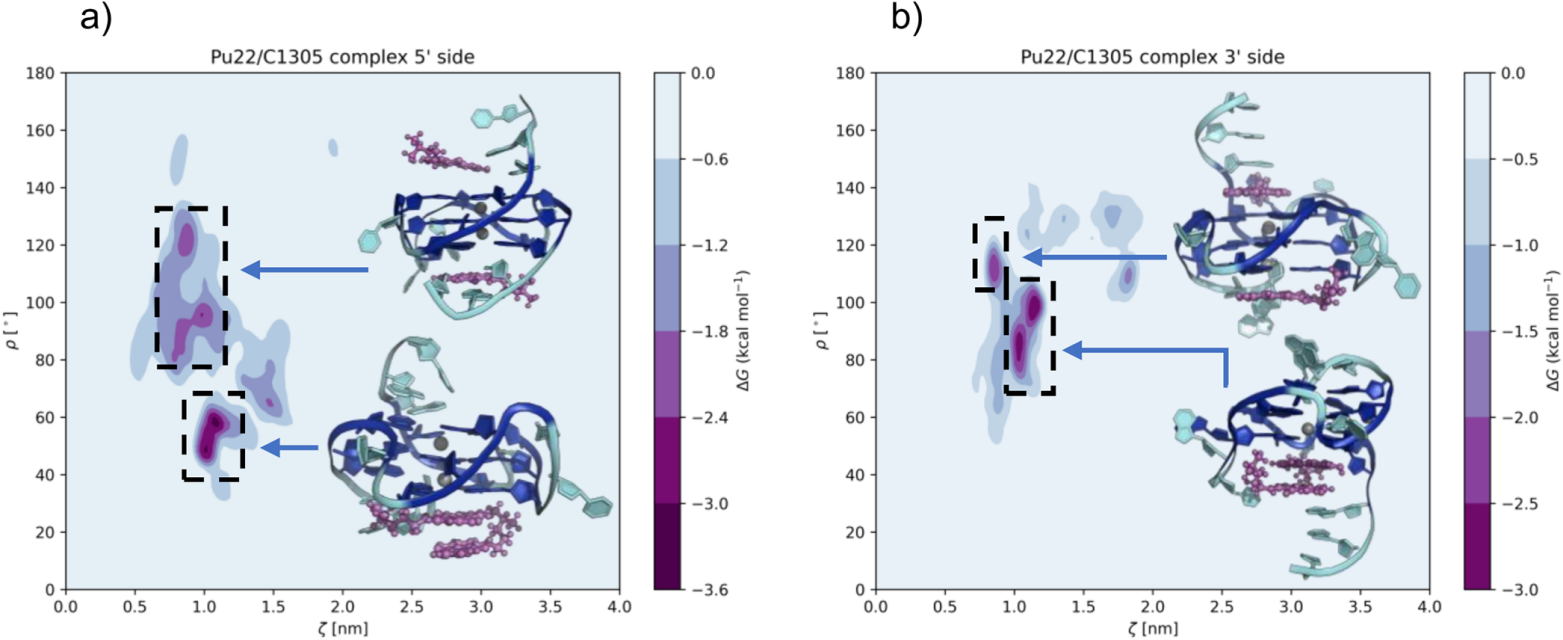
Free-energy landscapes for C-1305 interaction with Pu22 as a function of the ligand–G4 distance (ζ) and the horizon angle (ρ) reaction coordinates (RCs). a) The 5′-side complex: the landscape reveals two dominant free-energy minima (dashed boxes) corresponding to near-planar (ρ approximately 40–70°) and tilted (ρ approximately 80–130°) binding orientations. b) The 3′-side complex: exhibiting analogous minima that indicate a conserved, bidirectional recognition mechanism at both G-quadruplex termini. In both panels, representative snapshots illustrate the structures of the most populated clusters. (See Supporting Information for detailed RC definitions—Figures S12, S13 and Table S4).

### MTT Assay

Finally, we assessed whether this G4-targeting profile aligns with the reported antiproliferative activity of C-1305 in cells. Cell viability altered by the C-1305 compound was assessed using the MTT assay, which indirectly measures the number of live cells based on their ability to reduce MTT to purple formazan crystals in active mitochondria. The triazoloacridinone derivative exhibited potent cytotoxicity following 72 h incubation across multiple cancer cell lines, including those from pancreatic (Panc-1), lung (H460, A549), colon (HCT116), and breast (MDA-MB-231) cancers. IC_50_ values for C-1305 against these cells ranged from around 1.1 μM (H460, HCT116) to 2.15 μM (MIA PaCa-2), demonstrating high potency. Importantly, studies concerning normal cell lines, such as normal human lung fibroblasts MRC-5, normal human colon epithelial cells CCD 841 CoN, and immortalized human pancreatic ductal epithelial cells hTERT-HPNE,^44^ displayed markedly higher IC_50_ values of 7.1–9.0 μM, underscoring the selective toxicity of C-1305 toward malignant cells and suggesting a favorable therapeutic window (Table 1). As clinical references, two compounds were selected: cisplatin and doxorubicin. Cisplatin, a platinum-based chemotherapeutic standard for solid tumors including pancreatic, lung, colorectal, and breast cancers, shows IC_50_ values ranging from 3.4 to 51.2 μM in analogous MTT assays performed under comparable conditions in similar cell lines. The second drug, doxorubicin, a DNA intercalator and topoisomerase II inhibitor applied as a first-line chemotherapeutic agent against breast and lung cancer, exhibits lower IC_50_ values in the tested cancer cell lines, typically ranging from 0.1 to 0.46 μM. Taken together, these findings highlight the broad-spectrum antiproliferative efficacy of triazoloacridone C-1305, whose activity falls between that of cisplatin and doxorubicin, while maintaining a favorable degree of selectivity.

**Table 1:** Cytotoxicity of C-1305 against Human Cancer Cells

	Drug concentration, IC_50_[μM]		
		References	
Cell line	C-1305	Cisplatin	Doxorubicin
Panc-1	1.90 ± 0.40	3.4 ^31^	0.35 ^32^
MIA-PaCa-2	2.15 ± 0.37	51.2 ^31^	0.45 ^33^
H460	1.09 ± 0.09	4.5 ^34^	0.10 ^35^
A590	1.37 ± 0.09	16.8 ^36^	0.36 ^37^
HCT116	1.09 ± 0.32	12.0 ^38^	0.42 ^39^
MDA-MB-231	1.68 ± 0.36	7.8 ^40^	0.2 ^41^

## Conclusions

Taken together, in this work, we provide a consistent body of evidence suggesting that the strong “intercalative” binding of C-1305, reported in 2005 for guanine-triplet (GGG-containing) DNA sequences, may not necessarily reflect genuine insertion into duplex GGG tracts. Rather, we propose that at least some of the GGG-rich constructs examined at the time could plausibly populate G-quadruplex-like states in solution—an aspect that was difficult to fully anticipate or control given the limited contemporary awareness of G4 folding—and that the observed binding may, therefore, have originated from G4 engagement rather than duplex intercalation.^8^ Consistent with this interpretation, our dsDNA NMR data reveal no observable preference of C-1305 for intercalation into GGG motifs; instead, duplex intercalation is observed predominantly—almost selectively—at 5′-purine-pyrimidine-3′ dinucleotide steps, with a particularly strong preference for the TA/TA step.^9^ This sequence selectivity provides a clear framework for reconciling the earlier “GGG” binding phenotype with G4 engagement, while retaining the established capacity of C-1305 to interact with duplex DNA.

Our data further show that C-1305 forms a highly defined noncovalent complex with the c-MYC promoter G-quadruplex model Pu22, characterized by a DNA:ligand stoichiometry of 1:2, pronounced thermal stabilization of the folded G4 architecture, and a binding pattern dominated by interactions at the terminal guanine tetrads while largely sparing other regions of the quadruplex. Importantly, this G4 recognition mode can coexist with 1:2 binding stoichiometry, pointing to a dual interaction landscape that may be tunable by rational chemical design. From a ligand-design perspective, the triazoloacridinone aromatic core emerges as an attractive G4 pharmacophore—potentially more so than the related imidazoacridinone scaffold^7^—because it maintains a favorable and stable protonation state across a broad pH window, supporting translational relevance under near-physiological conditions. In line with this promise, C-1305 displays potent cytotoxic activity across a broad panel of cancer cell lines while being noticeably less toxic toward normal cells, corresponding to a several-fold selectivity window in the present data set. Given these properties and the progression of C-1305 to first-stage clinical evaluation for breast and colorectal cancers as well as leukemia, targeted optimization of this chemotype could represent a realistic path toward improved G-quadruplex selectivity. A particularly promising strategy is the deliberate redesign of the side chain: experience with unsymmetrical bisacridines indicates that sufficiently long and structurally elaborate side chains can mitigate dsDNA intercalation while preserving—and potentially enhancing—productive G4 engagement.^42,43^ Notably, initial efforts along these lines have already been undertaken and are yielding encouraging early outcomes.

## Supplementary Material



## Notes

Table of NMR chemical shifts of the protons of free Pu22 G-quadruplex and Pu22/C-1305 complex; table of couplings between Pu22 protons and C-1305 protons on the NOESY spectrum; regions of the two-dimensional metadynamics free-energy maps subjected to cluster analysis; anomeric–aromatic (H1′–H8), anomeric–anomeric (H1′–H2′/H2″), anomeric–anomeric (H1′–H3′) regions of the NOESY spectrum; NOE cross-peaks showing interactions between Pu22 and C-1305; UV–vis spectra of the p*K*_a_ series; residual spectra from PCA of UV–vis p*K*_a_ series; optimized tetrahedron; UV–vis spectra of the DNA/ligand complexation series; residual spectra from PCA of UV–vis complexation series; a depiction of ζ (distance) and ρ (horizontal angle); a depiction of ϕ (azimuthal angle); chemometric analysis—detailed information (PDF)

## Abbreviations

ATCC: American Type Culture Collection
COM: Center of mass
CV: Collective variables
DEPC: Diethyl pyrocarbonate
DMEM: Dulbecco’s Modified Eagle Medium
MD: Molecular Dynamics
FBS: Fetal Bovine Serum
NHE: Nuclease Hypersensitive Element
NMR: Nuclear Magnetic Resonance
NOE: Nuclear Overhauser Effect
NOESY: Nuclear Overhauser Effect Spectroscopy
NSCLC: Nonsmall Cell Lung Cancer
OD: Optical Density
PARP-1: Poly­(ADP-ribose) Polymerase-1
PCA: Principal Component Analysis
SPR: Surface Plasmon Resonance
SSE: Sum of Squared Errors
TASK: IT Center of the Tri-City Academic Computer Network
UV–vis: Ultraviolet–Visible Spectroscopy
