# Supplementary material for: Reframing Triazoloacridinone C‑1305 as a G‑Quadruplex Pharmacophore
Source: J Phys Chem B. 2026 May 11;130(20):5142–51. doi: 10.1021/acs.jpcb.6c01505 (PMC13309005; doi:10.1021/acs.jpcb.6c01505)
Supplement: Supplementary file 1 [file jp6c01505_si_001.pdf]

Supporting Information

Reframing Triazoloacridinone C-1305 as a G-Quadruplex Pharmacophore

Julia Pakuła[1], Julia Borzyszkowska-Bukowska[1], Monika Pawłowska[1], Rafał Tomaszczyk[1], Karolina Zielińska[2], Ewa Paluszkiewicz[1], Tomasz Laskowski[1]*

[1] Department of Pharmaceutical Technology and Biochemistry, Faculty of Chemistry, Gdańsk University of Technology, Gabriela Narutowicza Str. 11/12, 80-233 Gdańsk (Poland)

[2] Institute of Bioorganic Chemistry, Polish Academy of Sciences, Zygmunta Noskowskiego Str. 12/14, 61-704 Poznań (Poland)

Table S1. NMR chemical shifts δ[ppm] of the protons of free Pu22 G-quadruplex. 3
Table S2. NMR chemical shifts δ[ppm] of the protons of Pu22 G-quadruplex in Pu22/C-1305 complex 5
Table S3. Couplings between Pu22 protons and C-1305 protons on NOESY spectra. 7
Table S4. Regions of the two-dimensional metadynamics free-energy maps subjected to cluster analysis... 18

Figure S1. Anomeric-aromatic region (H1'/H8) of NOESY spectra of Pu22/C-1305 complex. .. 9
Figure S2. H1'-H2'/H2" region of NOESY spectra of Pu22/C-1305 complex. 10
Figure S3. H1'/H3' region of NOESY spectrum of Pu22/C-1305 complex................................ 11
Figure S4. NOE cross-peaks showing interactions between Pu22 G-quadruplex and C-1305. .. 12
Figure S5. ^{1}H-^{13}C HSQC spectrum of Pu22/C-1305 complex. 13
Figure S6. Second most populated state (as identified in the free-energy map).......................... 14
Figure S7. UV–Vis spectra of the pK_a series 14
Figure S8. Residual spectra obtained during the principal component analysis of UV-Vis pK_a series. 15
Figure S9. Optimized tetrahedron depicting four spectral forms, originating in the UV-Vis pK_a series. 15
Figure S10. UV–Vis spectra of the DNA/ligand complexation series 16
Figure S11. Residual spectra obtained during the principal component analysis of UV-Vis DNA/ligand complexation series.. 16
Figure S12. A depiction of ζ (distance) and ρ (horizontal angle) reaction coordinates.............. 17
Figure S13. A depiction of ϕ (azimuthal angle) reaction coordinate.. 17

Table S1. NMR chemical shifts δ[ppm] of the protons of free Pu22 G-quadruplex. Spectrum was recorded at 45ºC, D2O, 10mM potassium cacodylate buffer, pH=5.0, with presence of 10mM KCl

Residue	Proton type and δ [ppm]						
	H1’	H2’, H2’’	H3’	H4’	H5’, H5’’	H6/H8	CH_3
T4	6.090	2.092, 2.327	4.599	3.754	3.612, 3.612	7.512	1.799
G5	5.543	2.249, 2.338	4.593	3.955	3.614, 3.614	7.507	-
A6	5.762	2.320, 2.474	4.739	3.954	3.609, 3.777	7.859	-
G7	5.922	2.610, 2.871	4.868	4.157	3.946, 3.997	7.853	-
G8	6.012	2,483, 2.780	4.892	4.398	4.122, 4.149	7.552	-
G9	6.272	2.454, 2.600	4.997	4.470	4.153, 4.214	7.596	-
T10	6.383	2.349, 2.548	4.968	4.467	4.163, 4.201	7.740	1.852
G11	5.997	2.351, 2.772	4.966	4.322	4.116, 4.153	7.847	-
G12	6.005	2.529, 2.711	4.941	4.321	4.071, 4.152	7.759	-
G13	6.288	2.468, 2.614	4.918	4.360	4.148, 4.157	7.716	-
T14	5.683	1.528, 1.932	4.284	3.686	3.609, 3.621	7.093	1.541
A15	6.545	2.819, 2.949	5.053	4.446	4.067, 4.156	8.396	-
G16	6.031	2.505, 2.832	4.895	4.323	4.078, 4.119	7.955	-
G17	6.069	2.553, 2.836	4.896	4.409	4.170, 4.178	7.655	-
G18	6.312	2.479,2.578	5.002	4.485	4.119, 4.191	7.660	
T19	6.380	2.332, 2.532	4.982	4.468	4.163, 4.201	7.729	1.861
G20	5.868	2.237, 2.660	4.945	4.298	4.073, 4.178	7.600	-
G21	5.905	2.543, 2.591	4.939	4.382	4.041, 4.121	7.755	-
G22	6.051	2.412, 2.634	4.856	4.373	4.097, 4.171	7.474	-
T23	5.784	1.782, 2.218	4.621	4.052	3.943, 4.022	7.042	1.423
A24	5.666	1.926, 2.270	4.469	4.057	3.629, 3.846	7.683	-

A25	5.552	2.196, 2.088	4.271	3.756	3.385, 3.617	7.448	-

Table S2. NMR chemical shifts δ[ppm] of the protons of Pu22 G-quadruplex in Pu22/C-1305 complex. Spectrum was recorded at 45ºC, H2O/D2O 9:1 v/v, 10mM potassium cacodylate buffer, pH=5.0, with presence of 10mM KCl

Residue	Proton type and δ [ppm]						
	H1’	H2’, H2’’	H3’	H4’	H5’, H5’’	H6/H8	CH_3
T4	6.065	2.934, 2.073	4.526	3.713	3.543, 3.562	7.480	1.777
G5	5.725	2.848, 2.138	4.564	4.021	3.799, 3.819	7.813	-
A6	6.111	3.245, 2,410	4.804	4.089	3.825, 3.852	8.148	-
G7	5.839	3.408, 2.547	4.810	4.123	3.974, 3.989	7.812	-
G8	5.921	3.372, 2.428	4.819	4.351	4.107, 4.131	7.445	-
G9	6.227	3.210, 2.457	4.985	4.103	4.010, ?	7.525	-
T10	6.309	3.091, 2.244	4.861	4.347	4.123, ?	7.653	1.806
G11	5.903	3.296, 2.388	4.920	4.178	4.101, 4.101	7.686	-
G12	5.821	?, 2.470	4.786	4.338	4.001, 4.020	7.407	-
G13	5.900	2.831, 2.181	4.891	4.287	4.085, 4.152	7.672	-
T14	5.432	2.276, 1.124	5.426	4.111	3.164, 3.164	6.792	1.316
A15	6.522	3.542, 2.787	5.011	4.300	4.022, 4.092	8.412	-
G16	5.908	2.957, 2.309	4.773	4.172	3.985, 4.005	7.721	-
G17	5.798	3.185, 2.370	4.840	4.247	4.048, 4.065	7.505	-
G18	6.187	3.236, 2.529	4.890	4.299	4.071, 4.071	7.493	-
T19	6.441	3.130, 2.282	4.925	4.168	4.156, 4.156	7.683	1.825
G20	5.625	2.753, 2.054	4.809	3.766		7.305	-
G21	5.820	?, 2.149	4.899	?	4.005, 4.096	7.615	-
G22	6.036	3.228, 2.379	4.828	4.341	4.072, 4.139	7.393	-
T23	5.803	2.779, 1.855	4.674	4.021	3.944, 4.015	7.094	1.594

A24	These resonance could not be assigned unambiguously due to severe resonance superposition in this region of the spectrum.						-
A25	5.101	2.687, 2.036	4.436	3.806	3.466, 3.537	7.117	-

Table S3. Couplings between Pu22 protons and C-1305 protons on NOESY spectrum. Tetrads are numbered 5'->3', 5' tetrad consists of G7, G11, G16, G20, Middle tetrad consists of G8, G12, G17, G21 and 3' tetrad consists of G9, G13, G18, G22. L means loop between I and III tetrad, 5'SC means side chain from 5' side consists of T4, G5, A6, 3'SC means side chain from 3' side consists of T23, A24, A25.

Number of coupling	C-1305 proton type	Pu22 nucleotide	Pu22 proton type	Signal intensity	Element of G4
1	LH3	A6	H1'	Weak	5'SC
2	LH3	G20	H8	Medium	5' tetrad
3	LH3	G7	H2''	Strong	5' tetrad
4	LH7	T10	H2'	Weak	L
5	LH9	A6	H8	Medium	5'SC
6	LH9	G11	H2''	Medium	5' tetrad
7	LH9	G20	H1'	Medium	5' tetrad
8	LH9	A25	H8	Strong	3'SC
9	LH10	G18	H8	Medium	3'tetrad
10	LH10	T4	H2''	Strong	5'SC
11	LH10	A6	H3'	Strong	5'SC
12	LH10	G20	H1'	Strong	5'tetrad
13	LH15	T14	H2''	Weak	L
14	LH15	T23	CH_3	Medium	3'SC
15	LH15	G16	H1'	Medium	5' tetrad
16	LH15	G16	H2'	Weak	5' tetrad
17	LH15	G20	H2'	Weak	5' tetrad
18	LH16	A6	H8	Medium	5'SC

19	LH16	T14	H6	Weak	L
20	LH17	T4	H2''	Weak	5'SC
21	LH17	T10	CH_3	Weak	L
22	LH17	T23	CH_3	Weak	3'SC
23	LH18	A15	H1'	Medium	L
24	LH18	G16	H2'	Strong	5' tetrad
25	LH18	G16	H8	Medium	5' tetrad
26	LH18	G17	H1'	Medium	Middle tetrad
27	LH18	G12	H8	Strong	Middle tetrad

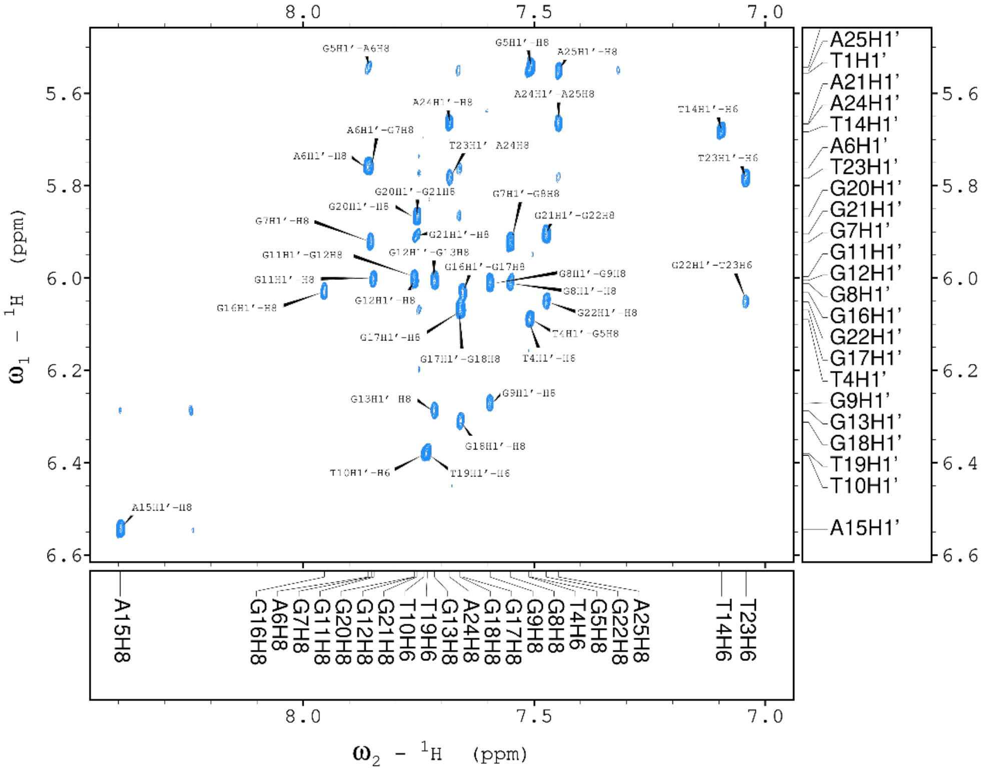

Figure S1. Anomeric-aromatic region (H1'/H8) of NOESY spectrum of Pu22/C-1305 complex. NMR spectrum was recorded on 700MHz spectrometer, 10mM KCl, 10mM potassium cacodylate buffer (pH=5.0) at 45ºC in D_2O.

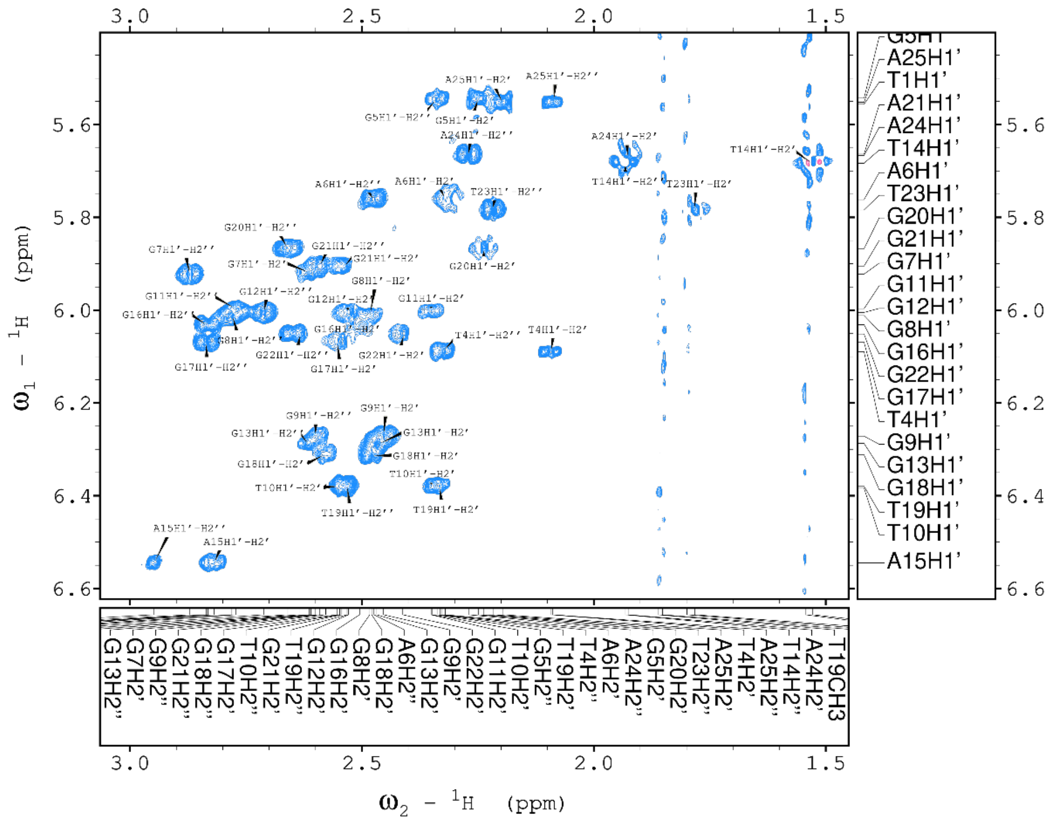

Figure S2. H1'-H2'/H2" region of NOESY spectrum of Pu22/C-1305 complex. NMR spectrum was recorded on 700MHz spectrometer, 10mM KCl, 10mM potassium cacodylate buffer (pH=5.0) at 45ºC in D2O.

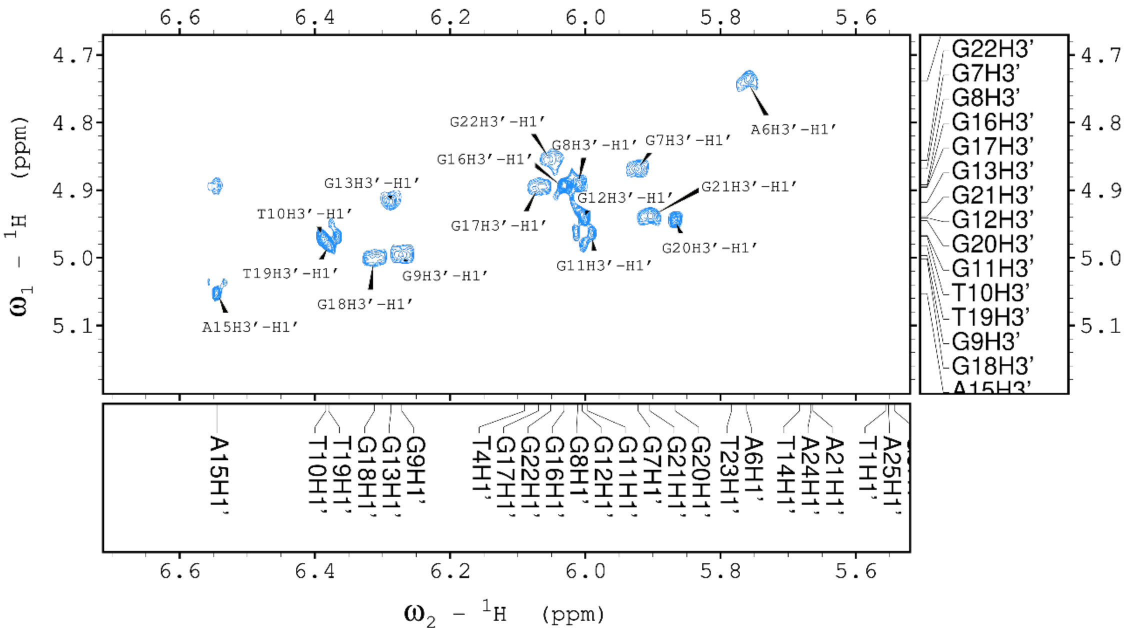

Figure S3. H1'/H3' region of NOESY spectrum of Pu22/C-1305 complex. NMR spectrum was recorded on 700MHz spectrometer, 10mM KCl, 10mM potassium cacodylate buffer(pH=5.0) at 45ºC in D2O.

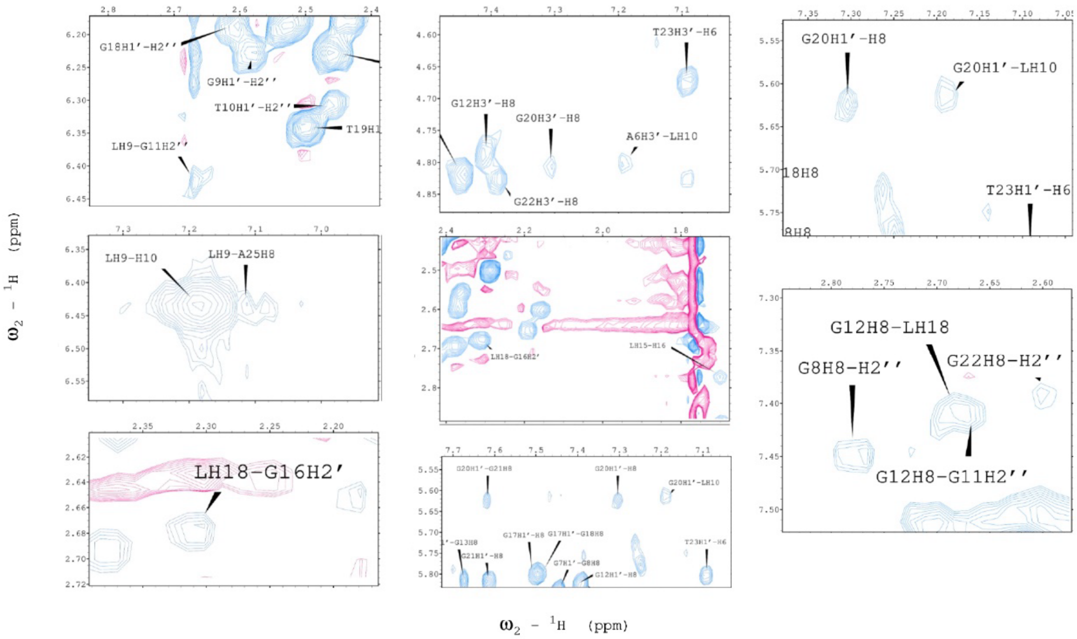

Figure S4. NOE cross-peaks showing interactions between Pu22 G-quadruplex and C-1305. View of different regions of the NOESY spectrum showing intermolecular interactions in Pu22/C-1305 complex. NMR spectrum was recorded on 700MHz spectrometer, 10mM KCl, 10mM potassium cacodylate buffer (pH=5.0) at 45ºC in D2O.

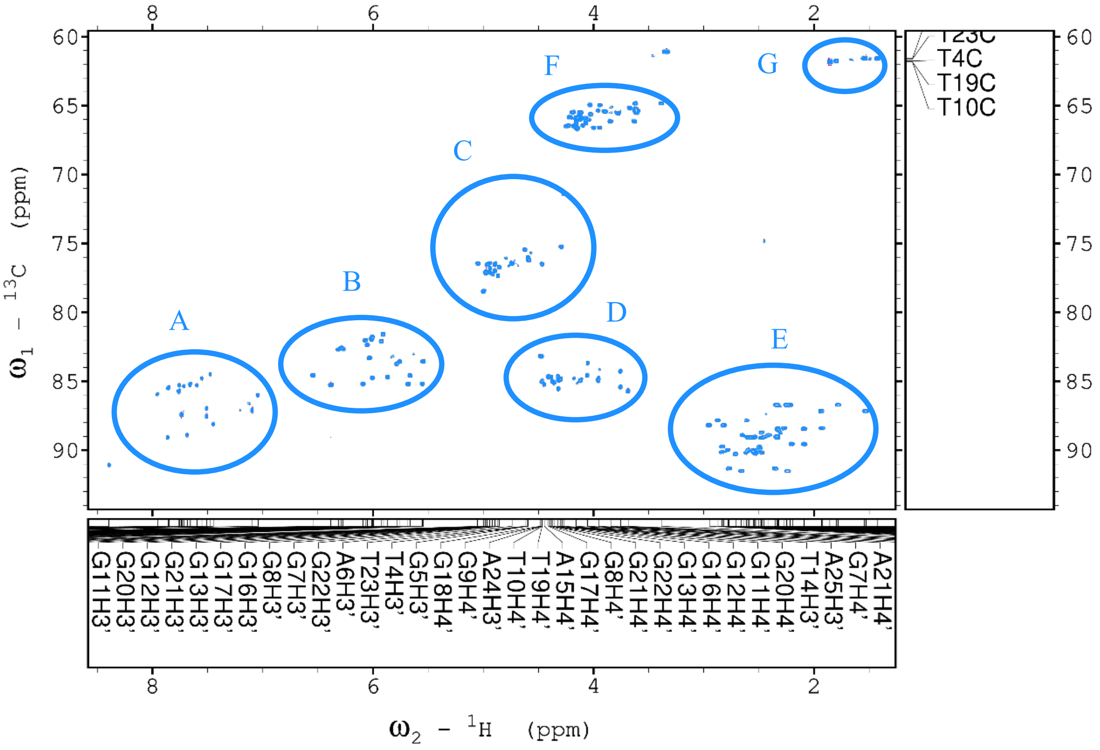

Figure S5. ^{1}H-^{13}C HSQC spectrum of Pu22/C-1305 complex. Couplings regions are shown on spectra: A)H6/H8-C6/C8, B)H1'-C1', C)H3'-C3', D)H4'-C4', E)H2'/H2''-C2', F)H5'/H5''-C5', G)methyl groups. NMR spectrum was recorded on 700MHz spectrometer, 10mM KCl, 10mM potassium cacodylate buffer (pH=5.0) at 45ºC in D2O.

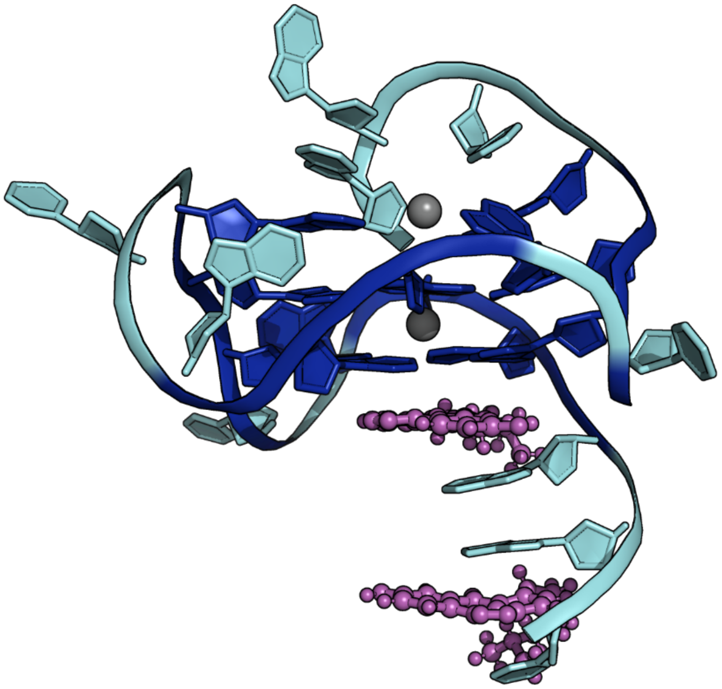

Figure S6. Second most populated state (as identified in the free-energy map shown in Fig. 4B, main manuscript): a local minimum located at ζ = 1.0–1.5 nm and ρ = 60–100°.

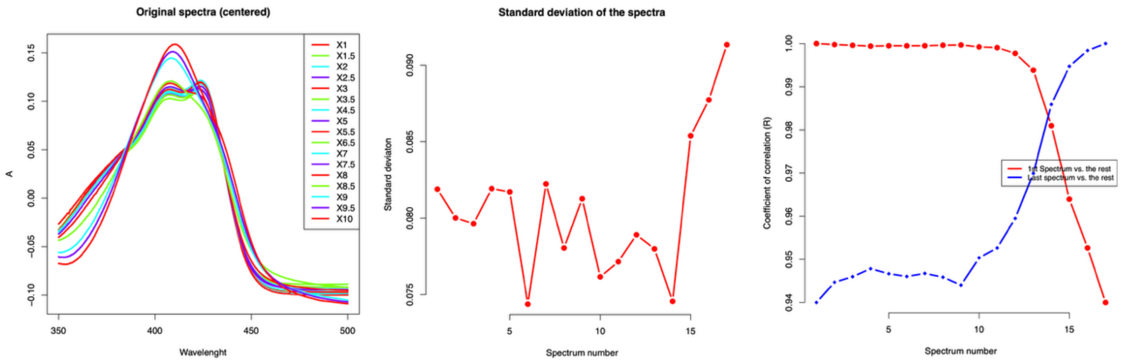

Figure S7. Centered raw UV–Vis spectra of the pK_a series (left), standard deviations of the centered spectra (middle), and correlation coefficients of the first spectrum (red trace) and the last spectrum (blue trace) with respect to all remaining spectra (right).

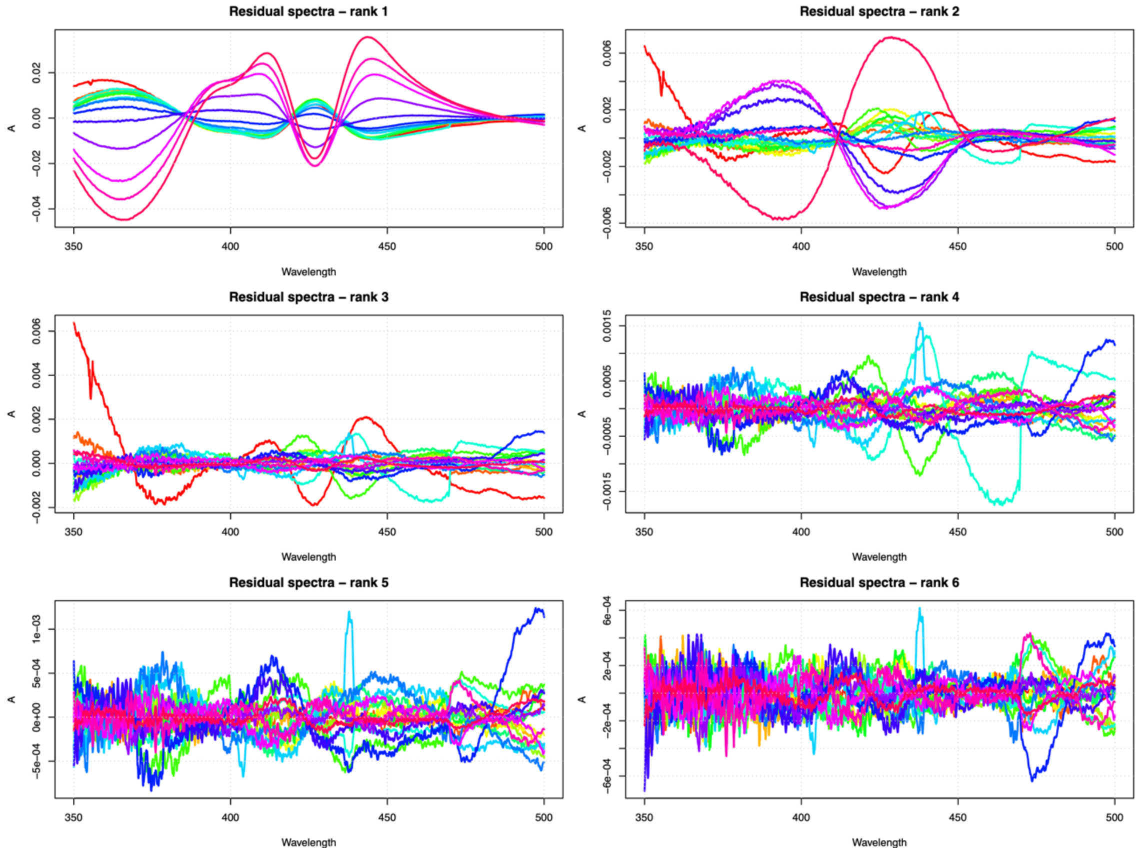

Figure S8. Residual spectra obtained during the principal component analysis of UV-Vis pK_a series.

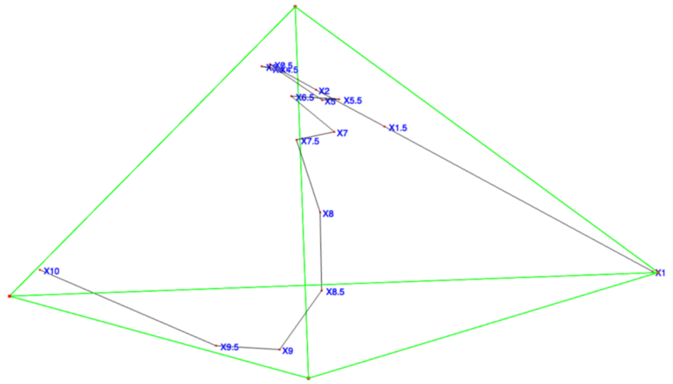

Figure S9. Optimized tetrahedron depicting four spectral forms, originating in the UV-Vis pK_a series.

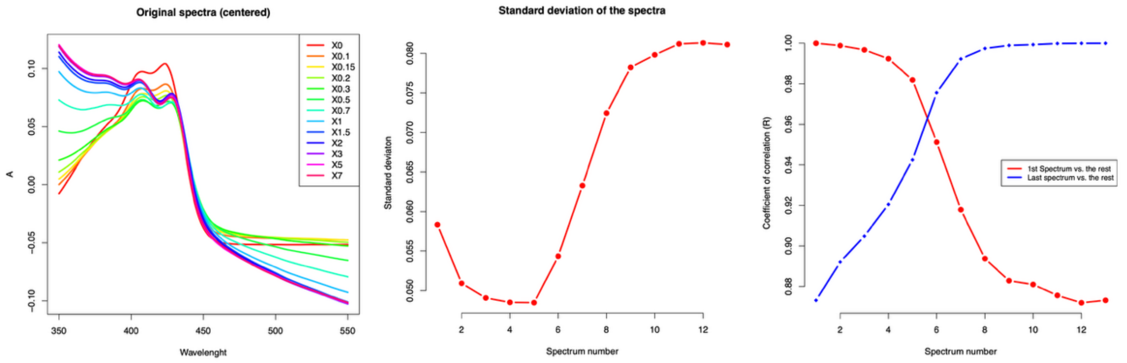

Figure S10. Centered raw UV–Vis spectra of the DNA/ligand complexation series (left), standard deviations of the centered spectra (middle), and correlation coefficients of the first spectrum (red trace) and the last spectrum (blue trace) with respect to all remaining spectra (right).

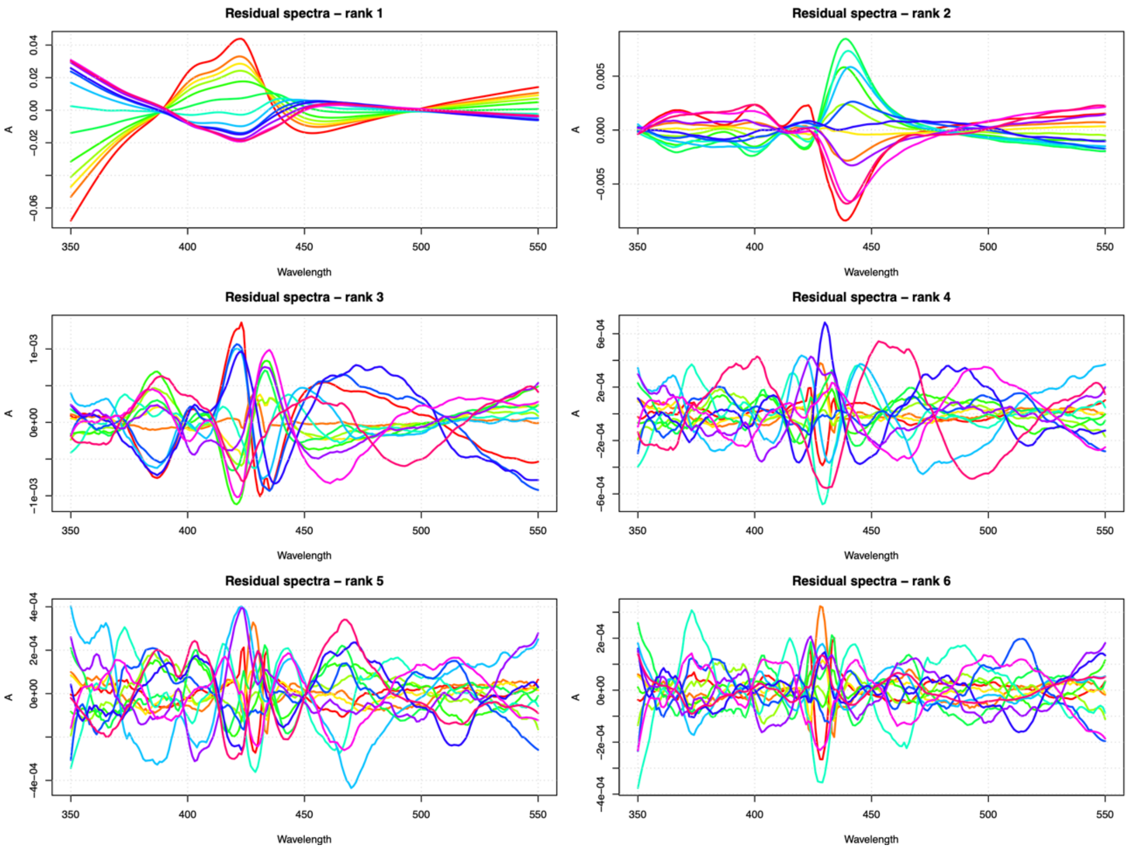

Figure S11. Residual spectra obtained during the principal component analysis of UV-Vis DNA/ligand complexation series.

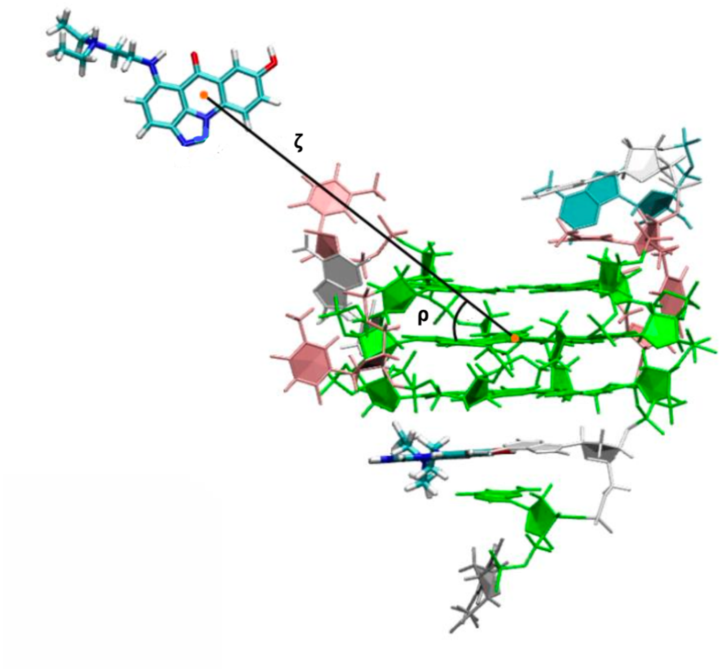

Figure S12. A depiction of ζ (distance) and ρ (horizontal angle) reaction coordinates.

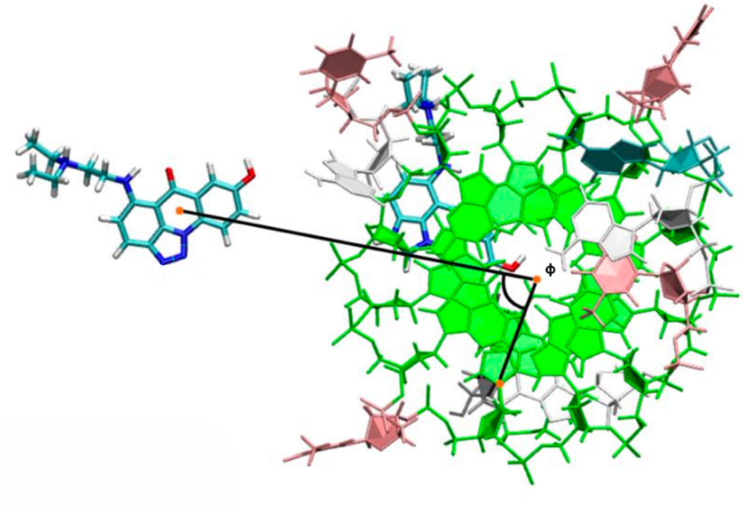

Figure S13. A depiction of ϕ (azimuthal angle) reaction coordinate.

Table S4. Regions of the two-dimensional metadynamics free-energy maps subjected to cluster analysis.

system	pair	p1_unit	p2_unit	p1_min	p1_max	p2_min	p2_max
5’-side	ζ - ρ	nm	rad	0.5	1.5	0.873	2.618
5’-side	ζ - ρ	nm	rad	0.8	1.5	0.0	0.873
3’-side	ζ - ρ	nm	rad	0.5	1.5	0.873	2.182
3’-side	ζ - ρ	nm	rad	1.5	2.0	1.309	2.618

CHEMOMETRIC ANALYSIS - DETAILED INFORMATION.

The procedure operates on a matrix of mean-centered spectra and exports diagnostic plots and intermediate matrices used later for identifying the effective number of spectral components (spectral forms).

Data representation used in our approach:

- probe: numeric matrix of centered spectra; rows correspond to wavelength points, columns correspond to individual spectra.
- rows: wavelength grid used for plotting (x-axis in spectral plots).
- n: number of spectra.

Basic diagnostics: variability and pairwise similarity.

For each centered spectrum i (column i of probe), the standard deviation s_i is computed and plotted as a diagnostic of spectral variability across the series. In addition, the workflow computes the covariance matrix between spectra and converts it to a correlation matrix to visualize similarity of each spectrum to the first and the last spectrum.

$$s_i = \mathrm{sd}(\mathbf{x_i}),\ \ \mathbf{x_i} = \text{column } i \text{ of } \mathbf{X}$$

$$\Sigma = \mathrm{cov}(\mathbf{X}) = \frac{1}{m-1}\,\mathbf{X^T}\cdot\mathbf{X}\quad (\text{here } m = \text{number of wavelength points/rows of } \mathbf{X})$$

$$\mathbf{R} = \mathrm{cov2cor}(\Sigma) = \mathbf{D^{-1/2}}\cdot\Sigma\cdot\mathbf{D^{-1/2}},\ \ \mathbf{D} = \mathrm{diag}(\Sigma_{11},\dots,\Sigma_{nn})$$

$$r_{ij} = \frac{\Sigma_{ij}}{\sqrt{\Sigma_{ii}\Sigma_{jj}}}$$

PCA by eigen-decomposition of the covariance matrix.

PCA is performed by eigen-decomposition of the covariance matrix Σ of the centered spectra. Eigenvalues (λ_i) and eigenvectors (principal directions, collected in V) are exported. Scores (principal component vectors for each spectrum) are obtained by projecting the data onto the eigenvector basis.

$$\Sigma = \mathbf{V} \cdot \mathbf{\Lambda} \cdot \mathbf{V}^{\mathbf{T}}, \;\; \mathbf{\Lambda} = \mathrm{diag}(\lambda_1, \dots, \lambda_n), \;\; \mathbf{V}^{\mathbf{T}} \cdot \mathbf{V} = \mathbf{I}$$

$$\mathbf{T} = \mathbf{XV}$$

$$\mathrm{VarExpl_i} = \frac{\lambda_i}{\sum_{j=1}^{n} \lambda_j}$$

Rank-k reconstruction and residual spectra.

To assess how many principal components are required to reproduce the experimental spectra, the workflow reconstructs spectra using the first k PCs (k = 1,2,...,n) and computes residual spectra as the difference between the original and reconstructed signals.

$$\mathbf{V_k} = [\mathbf{v_1}, \dots, \mathbf{v_k}], \;\; \mathbf{T_k} = \mathbf{XV_k}$$

$$\widehat{\mathbf{X}_k} = \mathbf{T_k} \cdot \mathbf{V_k^T} = \mathbf{X} \cdot \mathbf{V_k} \cdot \mathbf{V_k^T}$$

$$\mathbf{E_k} = \mathbf{X} - \widehat{\mathbf{X}_k} \quad \text{(residual spectra)}$$

A second PCA is performed on the selected loading vectors to obtain a low-dimensional representation of these vectors (Y) and visualize their relationships in a 2D map.

$$\mathbf{X_2} = \mathbf{V}_{\text{selected}}$$

$$\Sigma_2 = \mathrm{cov}(\mathbf{X_2}) = \mathbf{V_2} \cdot \mathbf{\Lambda_2} \cdot \mathbf{V_2^T}$$

$$\mathbf{T_2} = \mathbf{X_2} \cdot \mathbf{V_2}$$

$$\mathbf{Y} = \mathbf{X_2} \cdot \mathbf{V}_{\mathbf{2},(\mathbf{1}:\mathrm{dm})}$$

Geometric conversion of PCA outputs into fraction-like weights (bridge to equilibrium fitting).

To connect the PCA-based decomposition of UV–Vis spectra with subsequent equilibrium-model fitting (DNA–ligand binding and pKa analysis), we converted the PCA-derived spectral vectors into **fraction-like weights** using a simple geometric procedure in low-dimensional PCA space. In brief, each experimental spectrum corresponds to a point in a reduced PCA coordinate system. We then construct a minimal enclosing simplex (triangle in 2D or tetrahedron in 3D) that contains all points, and use **normalized distances to simplex facets** as coefficients that behave like non-negative weights and vary smoothly along the titration series. These weights are subsequently used as the input "ratios" matrix (columns = spectral forms) for downstream fitting.

Triangle enclosure (three-form case; 2D PCA map used in DNA/ligand complex formation).

In the three-form workflow, the PCA outputs are represented as 2D points (typically PC1 vs PC2). Our approach:

- plots the point cloud and defines an initial triangle (three vertices) that safely contains all points;
- optimizes the triangle geometry by random perturbations of the vertices, accepting only triangles that still enclose all points, and minimizing the triangle area. This yields a compact triangle that represents an empirical "endmember simplex" for the dataset;
- after obtaining the optimized triangle, the workflow computes, for each point (spectrum), the perpendicular distance to each triangle side.

These distances are normalized by the corresponding triangle heights, producing three non-negative coefficients per spectrum (one per side). The resulting 3-column matrix is exported as “ratios”, and visualized as “molar fractions” along the measurement order (e.g., pH series or titration step index).

This procedure yields smoothly varying, fraction-like coefficients that can be used directly as the experimental input for model fitting (binding or acid–base equilibria), while remaining agnostic about the exact microscopic identity of the underlying spectral states.

Tetrahedron enclosure (four-form case; 3D PCA map used in pKa determination).

In the four-form workflow, the PCA outputs are embedded in 3D (typically three PCs), and the goal is to obtain four fraction-like coefficients per spectrum. Our approach:

- initializes a symmetric tetrahedron and iteratively expands it until all experimental points lie inside;
- performs a multi-stage random-search optimization that perturbs tetrahedron vertices while maintaining the enclosure constraint, minimizing the tetrahedron volume to obtain a compact simplex around the data cloud;
- for each spectrum (point), calculates perpendicular distances to each of the four tetrahedron faces (planes defined by three vertices);
- normalizes these distances by the corresponding tetrahedron heights (distance from the opposite vertex to each face), yielding four non-negative coefficients per spectrum. The resulting matrix is exported as “ratios” and can be plotted along the experimental series;

For visualization and inspection, the script additionally generates interactive 3D renderings (via `rgl`) of the starting and optimized tetrahedra with the data points.

Interpretation and link to downstream fitting.

The simplex-derived coefficients are treated as empirical, fraction-like weights that quantify the position of each spectrum within the PCA-defined spectral manifold and provide a robust input for subsequent equilibrium modelling. Importantly, the simplex vertices should be understood as effective limiting spectral endmembers determined by the experimental trajectory and the enclosure constraint; they may not correspond to perfectly pure chemical species. Consequently, the resulting weights are used as a practical representation of the spectral evolution during titrations, enabling stable fitting of mechanistic models (DNA–ligand binding or pKa equilibria) in later stages of the computational workflow.

DNA:LIGAND COMPLEX FORMATION STUDIES.

Dilution model (titration in equivalents) in UV-VIS.

The experiment is parameterized by n = DNA equivalents added relative to the initial ligand amount. The initial ligand concentration is L_0 (in μM) at volume V_0. Each added equivalent increases the volume by a fixed fraction α of V_0 (α = 0.01 in the current dataset), so $V_{(n)} = V_0(1 + \alpha n)$. The total ligand amount is conserved, whereas DNA is added proportionally to n.

$$V(n) = V_0(1 + \alpha n)$$

$$L_t(n) = \frac{L_0}{1 + \alpha n}$$

$$D_t(n) = \frac{nL_0}{1 + \alpha n}$$

Omega (cooperativity) model: improved fit vs parameter stability.

Allowing ω to vary introduces an additional degree of freedom that can reduce the SSE and often provides a visibly better match in the transition region between predominantly free ligand and predominantly bound ligand. However, ω is typically strongly correlated with $K_{d,A}$ and $K_{d,B}$,

particularly when the experimental data resolve only three spectral components (free L, pooled DL, and DL_2). In such cases, multiple ($K_{d,A}$, $K_{d,B}$, ω) combinations may produce similar $\chi_{(n)}$ curves, leading to increased parameter uncertainty and reduced robustness in cross-validation.

In practical terms, the ω-fit model may overfit local features (or compensate for unmodelled species, e.g., minor ligand dimerization or weakly populated intermediates) and therefore can yield less stable parameter estimates under leave-one-out resampling, as reflected by larger LOO standard deviations. For this reason, the main text reports the conservative $\omega = 1$ model. Although the ω-fit model was thoroughly explored, its parameter estimates were typically less robust (higher LOO variability), and thus it was not adopted for reporting.

Computational fitting procedure.

The experimentally determined ligand-centered molar fractions (ratios) were provided as an N×3 matrix with titration points indexed by the DNA equivalents n. For robustness, the script automatically assigns the three columns to the expected spectral forms by inspecting which column is maximal at (i) the smallest n (assigned to free ligand), (ii) the largest n (assigned to the pooled singly-bound component $DL \equiv DL_A + DL_B$), and (iii) intermediate n (assigned to the doubly-bound component DL_2). The fractions were validated to sum to unity within numerical tolerance. Parameter robustness was assessed by leave-one-out (LOO) cross-validation. Each titration point was excluded in turn, parameters were refitted to the remaining data (with a reduced multistart budget), and the mean ± standard deviation of the resulting parameter estimates was reported.
